# Genome assemblies of the simultaneously hermaphroditic flatworms *Macrostomum cliftonense* and *Macrostomum hystrix*

**DOI:** 10.1093/g3journal/jkad149

**Published:** 2023-07-03

**Authors:** R Axel W Wiberg, Jeremias N Brand, Gudrun Viktorin, Jack O Mitchell, Christian Beisel, Lukas Schärer

**Affiliations:** Department of Environmental Sciences, Zoological Institute, University of Basel, Basel 4051, Switzerland; Department of Environmental Sciences, Zoological Institute, University of Basel, Basel 4051, Switzerland; Department of Environmental Sciences, Zoological Institute, University of Basel, Basel 4051, Switzerland; Department of Environmental Sciences, Zoological Institute, University of Basel, Basel 4051, Switzerland; Department of Biosystems Science and Engineering, ETH Zürich, Basel 4058, Switzerland; Department of Environmental Sciences, Zoological Institute, University of Basel, Basel 4051, Switzerland

**Keywords:** genome assembly and annotation, comparative genomics, *Macrostomum cliftonense*, *Macrostomum hystrix*, hermaphrodites

## Abstract

The free-living, simultaneously hermaphroditic flatworms of the genus *Macrostomum* are increasingly used as model systems in various contexts. In particular, *Macrostomum lignano*, the only species of this group with a published genome assembly, has emerged as a model for the study of regeneration, reproduction, and stem-cell function. However, challenges have emerged due to *M. lignano* being a hidden polyploid, having recently undergone whole-genome duplication and chromosome fusion events. This complex genome architecture presents a significant roadblock to the application of many modern genetic tools. Hence, additional genomic resources for this genus are needed. Here, we present such resources for *Macrostomum cliftonense* and *Macrostomum hystrix*, which represent the contrasting mating behaviors of reciprocal copulation and hypodermic insemination found in the genus. We use a combination of PacBio long-read sequencing and Illumina shot-gun sequencing, along with several RNA-Seq data sets, to assemble and annotate highly contiguous genomes for both species. The assemblies span ∼227 and ∼220 Mb and are represented by 399 and 42 contigs for *M. cliftonense* and *M. hystrix*, respectively. Furthermore, high BUSCO completeness (∼84–85%), low BUSCO duplication rates (8.3–6.2%), and low *k*-mer multiplicity indicate that these assemblies do not suffer from the same assembly ambiguities of the *M. lignano* genome assembly, which can be attributed to the complex karyology of this species. We also show that these resources, in combination with the prior resources from *M. lignano*, offer an excellent foundation for comparative genomic research in this group of organisms.

## Introduction

The free-living flatworms of the genus *Macrostomum* (Platyhelminthes) are simultaneous hermaphrodites, producing sperm and eggs at the same time. Several species of this genus are increasingly used as model systems in various contexts (Ladurner *et al*. 2005; Wudarski *et al*. 2020). In particular, *Macrostomum lignano* has become an important model species for research on diverse biological topics (Ladurner *et al*. 2005; Wudarski *et al*. 2020). This has involved the sequencing and assembly of the genome (Wasik *et al*. 2015; Wudarski *et al*. 2017), the development of transgenesis methods (Wudarski *et al*. 2017), and diverse gene expression studies to identify genes of interest in regeneration (Egger *et al*. 2006; Lengerer *et al*. 2018), reproduction (Arbore *et al*. 2015; Ramm *et al.* 2019; Brand *et al*. 2020; Wiberg *et al*. 2022), and stem-cell function (Ladurner *et al*. 2008; Grudniewska *et al*. 2016). However, the development of these resources uncovered challenges that could hinder the potential of *M. lignano* as a comprehensive genetic model organism. It was found that *M. lignano* is a hidden polyploid, having recently undergone whole-genome duplication and chromosome fusion events. Consequently, *M. lignano* has a set of large chromosomes absent in most other species (Zadesenets, Ershov, *et al*. 2017; Zadesenets, Schärer, *et al*. 2017). Moreover, the karyotype appears unstable, and substantial within-species karyotype polymorphisms were observed in *M. lignano* (Zadesenets *et al*. 2016; Wudarski *et al*. 2017) and 2 other species (Zadesenets *et al*. 2020). Such phenomena, while clearly of interest to researchers studying genome evolution, likely make the application of many modern genetic methods and tools more difficult (e.g. QTL mapping, GWAS, and genome editing).

Here, we present genome assemblies for 2 species of *Macrostomum*, namely *Macrostomum cliftonense* and *Macrostomum hystrix*, relatively close relatives of *M. lignano* (Brand, Viktorin, *et al*. 2022). These species have recently been proposed as complementary and genetically tractable model species (Schärer *et al*. 2020). A systematic survey of the karyotypes of several species of *Macrostomum* has found that both *M. cliftonense* and *M. hystrix* have 2*n* = 6 chromosomes, thought to be the ancestral state in this group, and that this is stable across >100 examined individuals for each species (Zadesenets *et al*. 2016, 2020). These 2 species also represent the 2 contrasting types of mating behavior, reciprocal copulation and hypodermic insemination, found in the genus (Schärer *et al*. 2011; Brand *et al*. 2022a). In the former, both partners insert their male copulatory organ into the partner's female storage organ in every mating and simultaneously donate and receive sperm. In the latter, individuals use a needle-like copulatory organ to inject sperm traumatically into their partner's tissues.

In addition to nuclear genome assemblies and annotations, we also establish other resources that have previously existed only for *M. lignano*, laying the foundation for exciting comparative analyses. In addition to building state-of-the-art gene models, we annotate transcripts with data from gene expression experiments that have proved valuable in *M. lignano*. In particular, we identify transcripts that are differentially expressed between adults and hatchlings (Brand *et al*. 2020) and transcripts with gene expression in different body regions containing reproductively interesting tissues and organs, i.e. positional RNA-Seq (Arbore *et al*. 2015). We also show how these annotations provide an excellent foundation for comparative research. Furthermore, we separately assemble and annotate mitochondrial genomes for both species. Our genome assemblies and the associated analyses greatly expand the resources available for this interesting group of organisms.

## Materials and methods

**Fig. 1.**
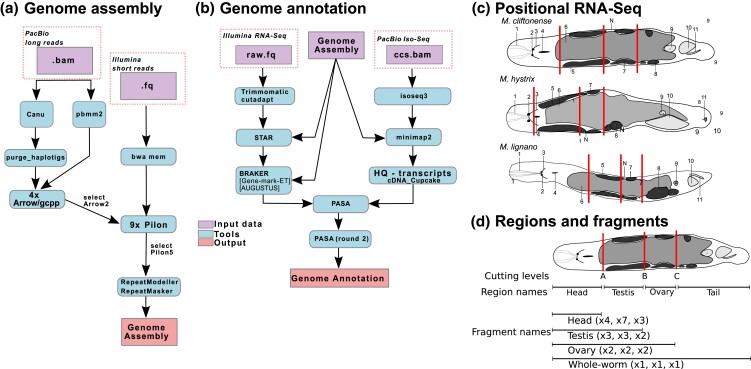
Overviews of a) the genome assembly and b) genome annotation pipelines showing the major steps. Independent sets of input data are highlighted in red dashed boxes. c) Diagrams of the anatomical organization of the studied *Macrostomum* species, with red vertical lines showing the cutting levels for each species used in the positional RNA-Seq study. Organs and internal structures are labeled as follows: (1) rhammites; (2) eyes; (3) neuropile; (4) mouth opening; (5) testis; (6) gut; (7) ovary; (8) developing eggs; (9) female antrum; (10) seminal vesicle; and (11) stylet. The “notch” between the testis and ovary is denoted as (N) and serves as a visual marker for cutting level B. d) Definitions of the cutting levels, region names, and fragment names are given for *M. cliftonense*. Cutting levels are represented by red vertical lines and are ordered “A,” “B,” “C” from anterior to posterior. The line segments indicate the parts of the worm defined as “regions” or “fragments” throughout the text. Since small fragments yield less RNA than large fragments, a large number of fragments were collected with a decreasing fragment size. Multipliers were based on the approximate relative length of each fragment compared with the whole worm and are shown in parenthesis next to the fragment names in the order *M. cliftonense*, *M. hystrix*, and *M. lignano*. Approximate final sample sizes for pools of each fragment are given in Supplementary Table 1c.

An overview of the bioinformatic pipeline for genome assembly and annotation is given in Fig. 1a and b. Complete details of the bioinformatic pipeline and the statistical analyses are provided in Supplementary Methods. Note that some references to methods, software, and algorithms only occur in the detailed methods in Supplementary Materials. A full table of sequencing data used in this study is provided in Supplementary Table 1.

### Species, culturing, and inbred lines

We establish novel genomic resources for 2 species of *Macrostomum*. The first species *M. cliftonense* Brand and Schärer, 2019, was recently described from Lake Clifton (South of Perth, Western Australia; Schärer *et al*. 2020) and is currently known only from this area. The species was initially named *M. cliftonensis* but was subsequently corrected to *M. cliftonense* (Zhang *et al*. 2021). The second species *M. hystrix* Örsted, 1843 sensu Luther 1905, is known from several European localities, ranging from the Baltic Sea to the Mediterranean (Giannakara and Ramm 2020; Schärer *et al*. 2020; Brand, Viktorin, *et al.* 2022). Note that the species name *M. hystrix* is ambiguous, as outlined in Schärer *et al*. (2011).

All worms were kept in glass petri dishes containing artificial seawater at species-specific concentrations and fed with diatoms cultured in *f*/2 culture medium (Supplementary Methods, Section 1.1). For *M. cliftonense*, we conducted 7–10 generations of full-sib inbreeding to obtain several inbred lines of reduced heterozygosity to facilitate genome assembly (Supplementary Materials, Section 1.2 and Supplementary Fig. 1). We finally used 1 of the 2 most fecund lines (GV23d; predicted inbreeding coefficient *F* ∼ 0.73) for sequencing and genome assembly. An inbred line was already available for *M. hystrix* (called SR1, predicted inbreeding coefficient *F* ∼ 0.998; Winkler and Ramm 2018), which we use here for the sequencing and assembly of the *M. hystrix* genome.

### gDNA, and RNA extraction and sequencing

We extracted gDNA of pooled worms and RNA from either pooled worms of different life stages, pooled worm fragments of different body regions, or whole individual worms (for details on sample collection, extraction methods, library generation, and sequencing, see Supplementary Materials, Section 2 and Supplementary Table 1). Sequencing libraries were in all cases generated by the Genomics Facility Basel (GFB, Department of Biosystems Science and Engineering, ETH Zürich in Basel), and sequencing was carried out by the GFB and the Lausanne Genomics Technologies Facility (University of Lausanne). In addition to generating new sequencing data, we also took advantage of publicly available reads and transcriptome assemblies from previous studies (Brand *et al*. 2020; Brand, Viktorin, *et al.* 2022; Wiberg *et al*. 2022; Supplementary Table 1). All raw sequencing data generated for this study were deposited in the European Nucleotide Archive under the BioProject accession PRJEB59187. Additional data files used in the analyses presented below are deposited in a Zenodo repository (https://doi.org/10.5281/zenodo.7861770). We also extracted and sequenced RNA from individual worms collected from the wild for *M. cliftonense* and *M. hystrix* (Supplementary Fig. 2), and from outbred laboratory cultures of *M. lignano*, permitting population genomic analyses that will be the focus of a forthcoming study.

### Genome assembly and annotation

The main steps of the assembly and annotation pipelines are shown in Fig. 1a and b (Supplementary Methods, Sections 3 and 4). In brief, we performed initial assemblies of PacBio reads with canu (Koren *et al*. 2017) and then evaluated for completeness and quality with BUSCO (Simão *et al*. 2015) and Merqury (Rhie *et al*. 2020). Due to the apparent assembly of separate haplotypes in both *M. cliftonense* and *M. hystrix*, we used Purge Haplotigs to remove redundant contigs representing these haplotypes (Roach *et al*. 2018; Supplementary Fig. 3). Illumina reads were preprocessed to remove adapters with Trimmomatic (Bolger *et al*. 2014). Subsequently, we performed several rounds of polishing using the PacBio long-read data, mapped with pbmm2 (from the pb-assembly suite: https://github.com/PacificBiosciences/pbbioconda), and Illumina short-read data, mapped with bwa mem (Li 2013), to correct assembly errors (Supplementary Fig. 4, and Supplementary Table 2a and b). Polishing was done with Arrow/gcpp (from the pb-assembly suite) for PacBio long-read data and pilon (Walker *et al*. 2014) for Illumina short-read data. We modeled and masked repeat elements with RepeatModeler (Smit and Hubley 2015) and RepeatMasker (Smit *et al*. 2015). We also performed these repeat modeling and masking steps for the available *M. lignano* genome to allow comparison, since repeats were analyzed differently in the original publication (Wudarski *et al*. 2017).

Next, we annotated the genome with transcriptome data (Illumina short-read and PacBio Iso-Seq; Supplementary Table 1Ab) from pools of adult and hatchling worms. We first identified and assembled spliced-leader (SL) sequences for *M. cliftonense* and *M. hystrix* from previously assembled transcriptomes (Supplementary Table 3), and removed SL sequences, along with TruSeq adapters, from Illumina RNA-Seq reads with cutadapt (Martin 2011). We then mapped the trimmed reads with STAR (Dobin *et al*. 2013) and used these as input to BRAKER (Hoff *et al*. 2016, 2019). We processed the PacBio Iso-Seq reads with the Iso-Seq3 and cDNA_Cupcake pipelines (from the pb-assembly suite). We then made a synthesis of the predicted gene models with PASA (Haas *et al*. 2003; Campbell *et al*. 2006; Haas 2011). We compared assembled annotations across *M. cliftonense*, *M. hystrix*, and the previously studied *M. lignano*, to identify orthologous genes between the 3 species (using the synteny-guided approach of Proteinortho/PoFF; Lechner *et al*. 2011, 2014).

We further annotated the assembled genes/transcripts using positional RNA-Seq data, to inform us about genes expressed in specific body regions of the worm containing reproduction-related tissues and organs (Fig. 1c and d; Supplementary Methods, Section 5.1 and Supplementary Tables 4–7). These results were validated for 1 annotation category based on published in situ hybridization (ISH) expression data from *M. lignano* (Supplementary Table 8). We also performed comparisons of expression in adult vs hatchling worms, to inform about genes that are more highly expressed in adults, as these are more likely to represent reproduction-related genes (since hatchlings are not reproductively active; Supplementary Table 9, and Supplementary Methods, Section 5.2).

Mitochondrial genomes were assembled from the Illumina short-read data using MITObim (Hahn *et al*. 2013), and annotated with the MITOS2 webserver (Bernt *et al*. 2013; Supplementary Methods, Section 6). Final genome assemblies and annotations were again evaluated for completeness and quality with BUSCO and Merqury, and we also present other standard genome assembly and annotation quality metrics in the results.

## Results and discussion

### Nuclear genome assembly and annotation

Here, we present annotated genome assemblies for 2 species of the flatworm genus *Macrostomum*, namely *M. cliftonense* and *M. hystrix* (Tables 1 and 2; Supplementary Table 2c). These resources will help develop alternative or complementary model systems to *M. lignano*, since the development of that species as a genetic model organism appears compromised by its recently discovered karyotype complexity (Schärer *et al*. 2020).

**Table 1.** Summary statistics for the assembly of the nuclear and mitochondrial genomes of 3 species of *Macrostomum*.

Species	*M. cliftonense*	*M. hystrix*	*M. lignano*
Reference	This study	This study	Wudarski *et al*. (2017)
**Nuclear genome**			
Total length	226,746,154	220,453,370	764,424,970
# Contigs	399	42	5,270
Mean contig length	568,286	5,248,890	145,052
N50 length	1,017,626	13,505,968	245,921
# L90	233	16	
Longest contig length	7,350,965	35,345,953	2,680,987
Shortest contig length	1,545	3,200	3,068
**Mitochondrial genome**			
Total length	15,813	20,472	14,192

All lengths are given in nucleotides. See Supplementary Table 2 for more details on assembly statistics.

**Table 2.** Summary statistics for the annotation of the nuclear and mitochondrial genomes in 3 species of *Macrostomum*.

Species	*M. cliftonense*	*M. hystrix*	*M. lignano*
Reference	This study	This study	Grudniewska *et al*. (2018)
**Nuclear genome**			
# Transcripts	73,177	54,873	90,195
Total transcript length	91,489,415	73,748,901	236,036,265
Mean transcript length	1,250	1,344	2,617
Longest transcript length	71,103	50,010	49,790
Shortest transcript length	9	60	8
# Genes	38,775	34,543	85,734
Total gene length	192,174,250	185,823,909	525,849,550
Mean gene length	4,956	5,380	6,133
Longest gene length	90,203	318,181	490,524
Shortest gene length	52	230	7
Mean # transcripts per gene	2	2	1
Mean # exons per gene	7	6	4
Mean exon length	858	788	721
Mean # introns per gene	5	5	3
Mean intron length	558	692	1,114
# (and %) SL *trans-*spliced genes			
10× coverage threshold	7,781 (20%)	7,254 (20%)	—
100× coverage threshold	5,550 (14%)	3,496 (10%)	—
# (and %) genes with multiple SL *trans-*splicing sites:			
10× coverage threshold	528 (1.4%)	341 (0.99%)	—
100× coverage threshold	153 (0.4%)	70 (0.2%)	—
**Mitochondrial genome**			
# MITOS2 annotated genes [tRNAs]	12 [21]	23 [22]	—

All lengths are given in nucleotides. See Supplementary Table 2, for more details.

**Fig. 2.**
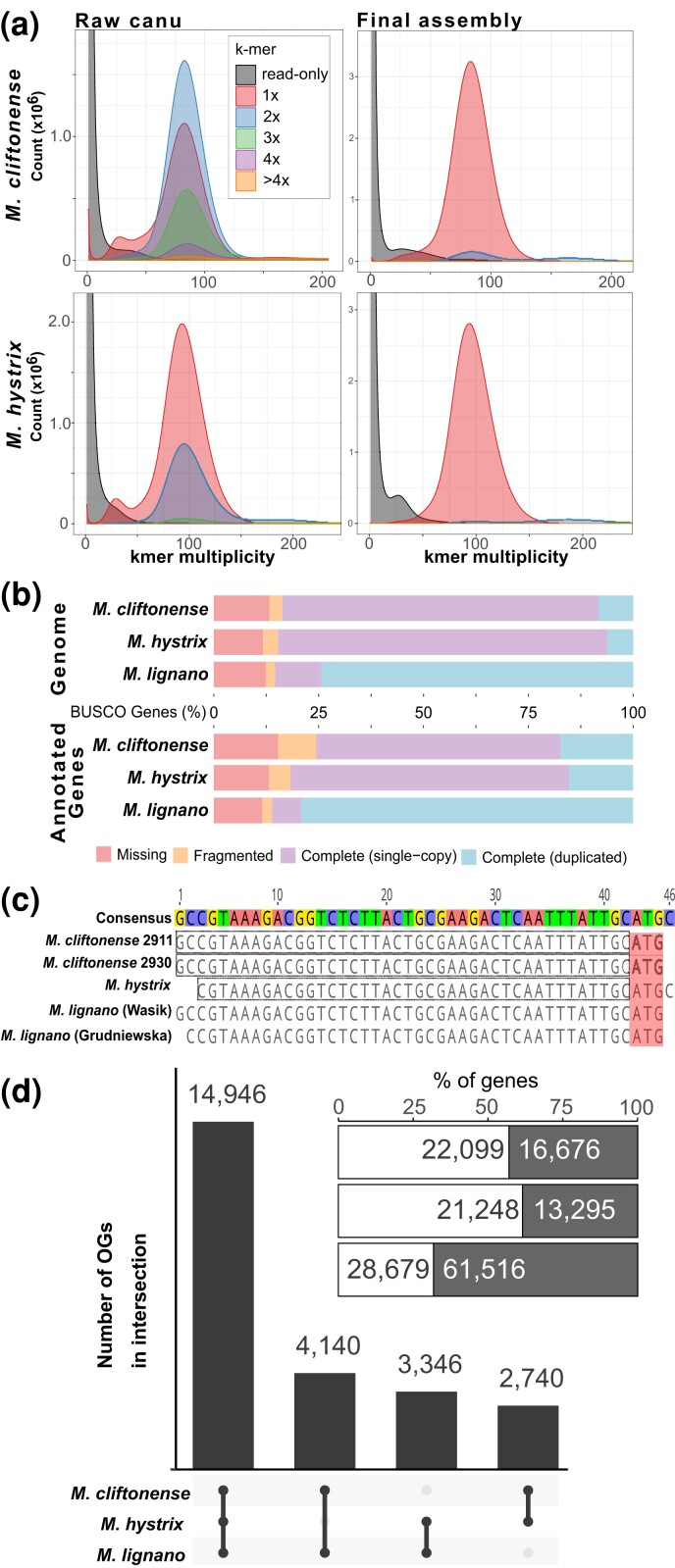
a) Merqury copy number spectrum plots of 19-mer multiplicity distributions from the initial canu assembly (left) and the final polished assembly (right) for *M. cliftonense* and *M. hystrix*. Colors indicate the number of 19-mer copies in the genome assembly, while the *x*-axis shows “multiplicity”—the number of 19-mer copies in the read-set. The gray distribution shows 19-mers that only exist in the read-set. b) BUSCO scores for the genome assembly and the annotated genes for the 2 species presented in this study, as well as the most recent assemblies for *M. lignano* (Mlig_3_7) for comparison. c) Alignment of assembled SL sequences from 2 samples of *M. cliftonense*, 1 *M. hystrix* sample, and 2 previously published sequences from *M. lignano*. The final sequences highlighted with dashed boxes were used for trimming (see the main text and Supplementary Methods, Section 3.6 for details; see also Supplementary Table 3). Note that the SL sequence likely contains the final ATG (translation start codon; highlighted in red after the dashed boxes), but this was not trimmed from the reads to avoid annotation problems for other genes that are not *trans*-spliced. d) The UpSet plot diagram showing the number of OGs containing representatives from each species. Note that the numbers include many-to-many OGs (not just one-to-one orthologs). The inset plot indicates the percentage, and absolute numbers of genes from each species, in the same order for in the UpSet plot, that were (white) or were not (gray) assigned to OGs.

The initial genome assemblies for *M. cliftonense* and *M. hystrix* were larger than expected based on flow-cytometry estimates (Schärer *et al*. 2020) and had high rates of duplication of BUSCO genes (see Supplementary Materials, Sections 3.1 and 3.2). Additionally, Merqury spectra-cn plots indicated that, especially for *M. cliftonense*, many *k*-mers present at high coverage among the reads were observed twice, or more, in the initial genome assembly (Fig. 2a). Given the well-documented stable karyotypes of 2*n* = 6 for both *M. cliftonense* and *M. hystrix* (Zadesenets *et al*. 2016, 2020), it is unlikely that karyotype polymorphisms within species or karyotype differences between species can explain these differences in *k*-mer spectra or larger than expected genome sizes. Instead, these results indicated separately assembled haplotypes, likely resulting from residual heterozygosity, so that multiple recombinant gene regions were sampled in the large pool of inbred worms from which gDNA was extracted.

After purging the contigs representing assembled haplotypes, the final assemblies still showed some residual duplication of some BUSCO genes, but the values were substantially lower than for the most recent *M. lignano* genome assembly (Fig. 2b). Moreover, Merqury spectra of the final assemblies showed that the vast majority of *k*-mers were now present only in single copies for both *M. cliftonense* and *M. hystrix* (Fig. 2a). Additionally, Merqury quality values (Rhie *et al*. 2020), N50, and L90 (the number of contigs containing up to 90% of the genome length) values were 36.5 and 42.8, ∼1 and ∼13 Mb, and 233 and 18 for *M. cliftonense* and *M. hystrix*, respectively (Table 1). The total proportion of the genome masked by RepeatMasker was 28, 22.9, and 32.3% across *M. cliftonense*, *M. hystrix*, and *M. lignano*, respectively (Supplementary Table 2d). These summary statistics indicate that the final assemblies for both *M. cliftonense* and *M. hystrix* are highly contiguous, largely haploid, and have high base pair accuracy. Moreover, these assemblies have far lower duplication rates of BUSCO genes than the most recent *M. lignano* assembly (Mlig_3_7; Wudarski *et al*. 2017), which was assembled into >5,000 contigs with an N50 of ∼245 kb. This likely reflects the simpler karyology of *M. cliftonense* and *M. hystrix* compared with *M. lignano* with its recent whole-genome duplications and karyotype instability (Zadesenets *et al*. 2016; Zadesenets, Ershov, *et al*. 2017; Zadesenets, Schärer, *et al*. 2017). The assembly for *M. hystrix* is generally of higher quality (e.g. higher contiguity and lower number of redundant haploid contigs), likely because the input material came from a more strongly inbred line. We also found, by an analysis of repeat content of all 3 species, that *M. cliftonense* and *M. hystrix*, have lower repeat content (28 and 22.9%, respectively), than *M. lignano* (32.3%). A full accounting of the nature of repeat content in these genomes will require more detailed work that is outside the scope of the current study.

### Gene annotation

The predicted gene and transcript content were similar for the *M. cliftonense* and *M. hystrix* genome assemblies with 38,775 (*n* = 73,177 transcripts) and 34,543 (*n* = 54,873 transcripts) annotated genes, respectively (Table 2). The number of annotated genes was more than 0.5× lower compared with *M. lignano* (Table 2). In total, 75.6 and 81.7% of conserved BUSCO genes were found among these annotated genes (Fig. 2b). Moreover, BUSCO duplication rates among annotated genes were much lower in *M. cliftonense* and *M. hystrix* (17.3 and 15.3%, respectively) than in *M. lignano* (79.1%). Once again, these results probably reflect the simpler karyology of *M. cliftonense* and *M. hystrix*, compared with *M. lignano*, and thus hint at their great potential as tractable genetic model systems.

We were also able to assemble SL sequences from both *M. cliftonense* and *M. hystrix* (Supplementary Table 3 and Fig. 2c). SL *trans*-splicing of genes is well known from Platyhelminthes (Rajkovic *et al*. 1990; Davis *et al*. 1995; Davis 1997; Zayas *et al*. 2005; Lasda and Blumenthal 2011), including *Macrostomum* (Wasik *et al*. 2015; Wudarski *et al*. 2017; Ustyantsev and Berezikov 2021). We found that the SL sequences themselves were highly conserved with respect to the previously identified SL sequence in *M. lignano* (Fig. 2c). Overall, we found evidence that 14–20 and 10–20% of genes were *trans*-spliced in *M. cliftonense* and *M. hystrix*, respectively, depending on the SL-containing read coverage threshold used (i.e. 100× or 10×; see Supplementary Methods, Section 4.2), and that between 153–528 (0.4–1.4%) and 70–341 (0.2–0.99%) annotated genes showed evidence for multiple SL locations (Table 2). This suggests that rates of mis-annotation of immature mRNAs from polycistronic genes are likely to be relatively low. Our upper estimates of the rates of SL *trans-*splicing (20 and 21% for *M. cliftonense* and *M. hystrix*, respectively) were somewhat lower, but approximately comparable with the most recent estimates from *M. lignano* (30%; Ustyantsev and Berezikov 2021). However, the significance of such variation in the rate of SL *trans*-splicing seems difficult to assess given the currently limited knowledge about variation described in other flatworms, with 3.2% in the planarian *Schmidtea mediterranea* (Zayas *et al*. 2005) and 20% in the trematode *Schistosoma mansoni* (Rajkovic *et al*. 1990; Davis *et al*. 1995). And even across studies of *M. lignano*, estimates depend greatly on the methodological approach, ranging from 11 to 30% (Wasik *et al*. 2015; Wudarski *et al*. 2017; Ustyantsev and Berezikov 2021). The new genome resources we present here thus provide an excellent starting point for better understanding SL *trans*-splicing in this interesting group of flatworms.

Genome-guided assembly of transcripts and gene models permitted a synteny-based ortholog detection approach. We identified a total of 25,172 orthogroups (OGs) with representative sequences from more than 1 species (Fig. 2d). In total, 59% of OGs had representative sequences from all 3 species (Fig. 2d). We found 9,479 one-to-one OGs (38% of all OGs), which permit straightforward comparative analyses. An additional 4,593 OGs (18%) had a single representative from *M. cliftonense* and *M. hystrix*, and 2 representatives from *M. lignano.* In contrast, only 117 and 138 OGs had 2 representatives from *M. cliftonense* or *M. hystrix*, suggesting an excess number of paralogs in *M. lignano.* These results, once again, likely reflect a signal of the previously mentioned whole-genome duplication of *M. lignano* compared with most other *Macrostomum* species (Wasik *et al*. 2015; Zadesenets *et al*. 2016, 2020; Zadesenets, Ershov, *et al*. 2017; Zadesenets, Schärer, *et al*. 2017).

### Positional RNA-Seq and adult vs hatchling contrasts

PCA of the raw positional expression data indicated good overall separation among fragments with the exception of whole worm (W) and ovary (O) fragments in *M. cliftonense* (Supplementary Fig. 5). Moreover, 3-dimensional scatterplots visualizing the fold changes between neighboring fragments (Supplementary Figs. 6–8) also showed that fairly similar data sets had been generated for each of the 3 species. For *M. cliftonense* and *M. hystrix*, positional annotations were assigned to 43,575 and 32,008 transcripts, respectively (Supplementary Table 5—Maccli and Machtx). Overall, the percentage of transcripts in each positional annotation category was similar between *M. cliftonense* and *M. hystrix*, but differed to some extent for *M. lignano.* This may again reflect annotation difficulties in *M. lignano* arising from the genome duplication (Supplementary Table 6; Zadesenets, Ershov, *et al*. 2017; Zadesenets, Schärer, *et al*. 2017).

The prior positional RNA-Seq analysis of Brand *et al*. (2020), which used only a single pool of RNA-Seq data for each fragment (from Arbore *et al*. 2015), was performed with the same *M. lignano* assembly as here. We could therefore compare the agreement of annotations for all transcripts that were annotated in both studies. Overall, of the 51,886 transcripts that were annotated in both studies, 40,627 (77.4%) received the same annotation (Supplementary Table 7). The largest changes were between the reproduction-related annotation categories (testis, ovary, and tail) and the nonspecific and other classifications. Despite these substantial deviations across studies, the comparison of the new annotations for *M. lignano* to expression patterns found in ISH experiments demonstrates that the updated positional RNA-Seq data more precisely detects tail-region transcripts. Of the transcripts bioinformatically annotated as tail-region specific, for which ISH experiments had also been performed, 59% showed ISH patterns that could be described as tail-region specific (Supplementary Table 8). In contrast, this percentage was only 54% in the original study by Brand *et al*. (2020). Based on this improvement in the agreement between ISH patterns compared to the previous positional RNA-Seq studies in *M. lignano* (Arbore *et al*. 2015; Brand *et al*. 2020), we are confident that the positional RNA-Seq data we generated for *M. cliftonense* and *M. hystrix* are also informative about reproduction-related transcripts, even in the absence of further ISH validation experiments for these species.

Additionally, we used data from pools of adults and hatchlings to categorize transcripts into those with higher expression in adult worms vs hatchlings (Supplementary Table 9), allowing further refinement of the categorization of genes as “reproduction related.” Patterns of adult and hatchling expression appear quite similar across all 3 species. The proportion of transcripts that were differentially expressed in adult vs hatchling worms varied between 44 and 23.7% in *M. cliftonense* and *M. hystrix*, respectively (Supplementary Fig. 9a). There were always more transcripts with higher expression in adults (Up-in-A) than with higher expression in hatchlings (Up-in-H). Reproduction-related transcripts (i.e. those annotated as expressed in the testis, ovary, or tail region) are significantly more likely to have higher expression in adults than expected based on the proportion of all analyzed transcripts in each reproduction-related annotation category (Supplementary Fig. 9b). Reproduction-related categories (i.e. testis, ovary, and tail region) therefore largely represent transcripts that have higher expression in reproductively active adults, which is expected if these transcripts are indeed primarily reproduction related. In addition, we observe a similar group of transcripts with much higher expression in adults compared with hatchlings, as also seen in a previous study in 2 additional *Macrostomum* species (see Brand *et al*. 2020; Supplementary Fig. 9).

We also annotated the synteny-based OGs with the expression patterns of each species. We were thereby able to identify OGs with conserved reproduction-related functions across species. However, the overlap in positional annotations across all 3 species was relatively low, even when allowing for some difficulties in annotation due to duplications present in *M. lignano*. These results may point to a high turnover of genes with reproduction-related function, such that functions are not typically conserved across somewhat diverged species for these rapidly evolving genes (for the respective phylogenetic placements, see Brand, Viktorin, *et al*. 2022). Alternatively, reproduction-related genes may be difficult to annotate reliably. On the one hand, they require accurate gene expression information from different body regions and tissues, which is technically challenging. We note, for example, that, as a result of anatomical differences, homologous fragments from across the worms are somewhat differently sized (Fig. 1c). This could result in variation across species in the ability to detect gene expression differences between neighboring fragments. On the other hand, even with synteny-guided approaches, homologous sequences remain challenging to detect for rapidly evolving genes, making them difficult to assign to OGs (so-called “homology detection failure”; Weisman *et al.* 2020; Wiberg *et al*. 2022).

From the distribution of annotation categories across OGs, we found that those with tail-region annotations are particularly rare, with at most 13 OGs using the most liberal definition (Supplementary Table 10), perhaps reflecting variation in gene turnover or evolutionary rates across annotation groups. A substantial number of testis-region annotated OGs are identified, as well as a large number of OGs with nonspecific annotations (Supplementary Table 10). Overall, there were fewer OGs with reproduction-related positional annotations than with nonspecific annotations, regardless of whether we used the strict, relaxed, or liberal OG definition (Supplementary Table 10). Based on the proportions of reproduction-related annotations within each species, there were always fewer OGs with ovary- and tail-region annotations than expected, perhaps reflecting particularly rapid sequence evolution (χ^2^ tests, all *P* < 0.001; Supplementary Table 10). Surprisingly, testis-region annotations were more commonly observed than expected based on their distributions in *M. cliftonense* and *M. lignano*, but not in *M. hystrix*. This may reflect the changes in selection pressures from altered mating strategies in the hypodermically inseminating species, *M. hystrix*. If some transcripts are no longer required for producing certain phenotypes, such as the sperm bristles characteristic of reciprocally copulating species (Schärer *et al*. 2011; Brand *et al*. 2022a), then the functions and expression patterns of these transcripts may be more free to change, resulting in divergent expression patterns across species (Wiberg *et al*. 2022). In this context, OGs with testis-region annotations for *M. cliftonense* and *M. lignano*, but not *M. hystrix* or other hypodermically inseminating species, may be promising candidates for identifying genes underlying sperm bristle development or other sperm-related traits (Brand *et al*. 2022a; Wiberg *et al*. 2022). Although we stress that we currently only have a small sample size (2 reciprocally copulating and 1 hypodermically inseminating species) from which to draw such comparative conclusions, these observations therefore merit further investigation.

We found that 12.4% of the strict one-to-one OGs (OGs with a single representative sequence from each species) had the same patterns of differential expression in adult vs hatchling worms across species (Supplementary Fig. 9c). Specifically, among one-to-one OGs with consistent annotations across species, we found a highly significant association between the positional annotation categories and whether expression was higher among adults or hatchlings (*χ*^2^ = 1,043.8, d.f. = 6, *P* < 0.001). OGs labeled as having reliably higher expression in adults for all species were highly enriched for reproduction-related annotation categories (Supplementary Table 11), suggesting that they are primarily expressed in reproductively active worms. Meanwhile, OGs of transcripts with no detectable difference in expression between adults and hatchlings were more likely to also be annotated as nonspecific in expression throughout the worm, across all species (Supplementary Table 11). These annotated OGs thus provide an excellent vantage point from which to study the evolution of reproduction-related genes in this genus.

### Mitochondrial genome assembly

In addition to the nuclear genomes, we also assembled and annotated the mitochondrial genomes for both *M. cliftonense* and *M. hystrix* (see Supplementary Methods, Section 6). The mitochondrial assembly was marginally longer for *M. cliftonense* compared with *M. lignano*, while that of *M. hystrix* was substantially longer (Table 1). In addition, the number and complement of annotated genes and tRNAs differed from a prior survey of the *M. lignano* mitochondrial genome (Table 2; Supplementary Table 2e; see Egger *et al*. 2017). MITOS2 annotated 21 tRNAs in *M. cliftonense* and 22 in *M. hystrix* (including an apparent duplication of tRNA C). *Macrostomum hystrix* apparently lacks an L1 tRNA, as is the case in *M. lignano* (Egger *et al*. 2017). In contrast, *M. cliftonense* lacks the H and Y tRNAs but has an additional copy of the C tRNA. Additionally, the order of genes and tRNAs is not conserved across the 3 species (Supplementary Table 2e). Interestingly, in *M. hystrix*, it was recently reported that the cytochrome c oxidase I (COI) gene contains a single base deletion, resulting in a frameshift mutation and a premature stop codon (Schärer *et al*. 2020). The previously published COI sequences from *M. hystrix* achieved blast hits with 100% identity and coverage on the assembled mitochondrial genomes, thereby independently confirming this frameshift mutation. In this context, we also note that the automated MITOS2 annotation of the COI locus is split for *M. hystrix*, but not *M. cliftonense*, in the region of this frameshift mutation (Supplementary Fig. 10, and Supplementary Table 2e). It is also noteworthy that the larger total number of genes that MITOS2 identified in *M. hystrix* is primarily due to a large number of gene annotations being split into 2 or more parts in the same way as for COI. Together, these findings point to potentially many more structural mutations in the mitochondrial genome of *M. hystrix*, and potentially other closely related *Macrostomum* species (Shi *et al*. 2022). These interesting observations merit further investigation.

In conclusion, in this study, we have assembled and annotated novel genomes for 2 *Macrostomum* species, *M. cliftonense* and *M. hystrix*, along with functional information in the form of positional RNA-Seq experiments and adult vs hatchling gene expression contrasts. These genetic resources will help develop these species as complementary model systems to the well-established *M. lignano*, where genetic and karyotypic complexities could prevent the efficient application of modern genetic methods and tools. We show that these genomes and the associated sequencing data sets provide an excellent foundation from which to begin comparative investigations within this genus.

## Supplementary Material

jkad149_Supplementary_Data

## Data Availability

All raw sequencing data generated for this study were deposited in the European Nucleotide Archive (ENA) under the BioProject accession PRJEB59187 (see also Supplementary Table 1). The *M. cliftonense and M. hystrix* genome assemblies are deposited in ENA under the same BioProject with the accession numbers: GCA_950096795 and GCA_950097015. Annotation files in GFF3 format, as well as other additional files and scripts used in the analyses presented, are deposited in a Zenodo repository (https://doi.org/10.5281/zenodo.7861770). Genomes and annotations will also be hosted in an upcoming release of WormBase ParaSite (parasite.wormbase.org). Supplemental material available at G3 online.
