# Supplementary material for: Genome assemblies of the simultaneously hermaphroditic flatworms Macrostomum cliftonense and Macrostomum hystrix
Source: G3 (Bethesda). 2023 Jul 3;13(9):jkad149. doi: 10.1093/g3journal/jkad149 (PMC10468722; doi:10.1093/g3journal/jkad149)
Supplement: jkad149_Supplementary_Data [file jkad149_supplementary_data.zip › Supplemental_Text,_Figures,_and_Tables_G3-2023-404251.pdf]

Genome assemblies of the simultaneously hermaphroditics flatworms *Macrostomum cliftonense* and *Macrostomum hystrix*

Wiberg et al.

Supplementary materials

Supplementary methods

1. Culture maintenance and inbred lines

1.1 Culture maintenance

1.2 Inbred lines

2. DNA and RNA extraction, and sequencing

2.1. gDNA extraction and sequencing

2.2 RNA extraction and sequencing: genome annotation and adult vs. hatchling contrasts

2.3 RNA extraction and sequencing: positional RNA-Seq datasets

2.4 RNA extraction and sequencing: population samples

3. Genome assembly

3.1 Initial genome assembly – M. cliftonense

3.2 Initial genome assembly – M. hystrix

3.3 Polishing of genome assemblies

3.5 Identification and masking of repeat regions

4. Genome annotation

4.1 Identification of spliced leader (SL) sequences

4.2 Illumina RNA-Seq data

4.3 PacBio Iso-Seq data

4.4 Data integration and detecting trans-spliced genes

4.5 Ortholog identification

5. Differential gene expression analyses

5.1 Positional RNA-Seq

5.2 Adult vs. hatchling RNA-Seq data analysis

6. Assembly and annotation of the mitochondrial genome

1. Culture maintenance and inbred lines

1.1 Culture maintenance

All worms were kept in kept in glass petri dishes containing artificial seawater (ASW) made of distilled water and artificial sea salt (hw-Marinemix professional, Wiegandt, Germany) at species-specific concentrations (*M. cliftonense*: 32 ‰; *M. hystrix*: 6 ‰). Worms were fed with diatoms (*Nitzschia curvilineata*) cultured in f/2 culture medium at 32‰ (Andersen et al. 2005).

1.2 Inbred lines

To generate inbred lines of *M. cliftonense* that capture much of the available genetic diversity, we combined juvenile individuals from eight different dishes of our outbred lab culture into 24 parental pairs (figure S1). After the pairs had begun to reproduce, we separated each pair into individuals “a” and “b” to lay clutches of eggs (figure S1). Since only some parent individuals produced offspring after separation, we combined them once more into the same pairs. Once separated a second time individuals were now named “c” and “d” (because it was not possible to identify the original “a” and “b” individuals after pairing them; figure S1). From the resulting F1 offspring of each individual we set up full-sib pairs for further inbreeding, taking only offspring from the same clutch (“intra-clutch full-sibs”). From these F1 offspring, we then continued with a full-sib inbreeding procedure for 7 to 10 generations, depending on the resulting fecundity and generation time (figure S1). Finally, we chose one pair to establish a mass culture from all resulting offspring (as well as the pair itself). In this manner we succeeded in establishing cultures of a total of eight inbred lines from single F7-F10 offspring pairs (figure S1). For the most fecund line, derived from parent individual 23b, two parallel cultures GV23c and GV23d were established from two full-sib F7 pairs as a backup against loss. Note that while the other lines are derived from separate initial pairs and are thus independent, lines GV23c and GV23d share direct grand-parents (figure S1).

An inbred line was already available for *M. hystrix* (called SR1, predicted inbreeding coefficient $F \sim 0.998$; Winkler & Ramm 2018). We used that line here for the sequencing and assembly of the *M. hystrix* genome, and the same line has previously been used in other studies on this species (e.g., Giannakara & Ramm 2020; Brand et al. 2020).

2. DNA and RNA extraction, and sequencing

2.1. gDNA extraction and sequencing

High molecular weight genomic DNA (HMW gDNA) was extracted from pooled worms isolated from cultures of inbred lines GV23d (*M. cliftonense*, ~900 worms) and SR1 (*M. hystrix*, ~700 worms) at various stages of development (i.e. juveniles, subadults and adults). Worms were treated with an antibiotic mix of Rifampicin (from 50 mg/ml stock in DMSO), Ampicillin, Neomycin, Streptomycin, and Spectinomycin (each from 50 mg/ml stock in water, for a final concentration of 50 µg/ml of each component), and starved for 6 days prior to gDNA extraction, transferring the worms to new antibiotic containing medium on the third day. HMW gDNA was extracted, following a CTAB de-mucusing step (Grohme et al. 2018), using a phenol-chloroform extraction protocol (following Wasik et al. 2015). PacBio SMRTbell sequencing libraries were prepared by the Genomics Facility Basel (GFB, Department of Biosystems Science and Engineering, ETH Zürich in Basel). Blue Pippin size selection was conducted to remove fragments < 20 kb. PacBio sequencing of HMW gDNA libraries was performed on the Sequel platform (Pacific Biosciences, Menlo Park, CA, USA) at the GFB with two 1M SMRT cells per species. For *M. cliftonense* and *M. hystrix* we obtained a total of ~800k and ~350k reads, respectively, representing coverages of ~70x and ~44x of the respective genomes (table S1Aa).

For short-read genome sequencing of the inbred lines, gDNA was extracted from inbred lines GV23d and GV23c (*M. cliftonense*), and SR1 (*M. hystrix*) using the DNEasy Kit (QIAGEN, Hilden, Germany). Illumina libraries were prepared at the GFB using the KAPA HyperPrep kit (Roche, Basel, Switzerland). Libraries were sequenced on one lane on the NovaSeq 6000 platform at the GFB for a total coverage of ~100x and ~130x for *M. cliftonense* and *M. hystrix,* respectively (table S1Aa).

2.2 RNA extraction and sequencing: genome annotation and adult vs. hatchling contrasts

For *M. cliftonense*, we generated 8 samples of pooled worms; 4 adult pools (>2 weeks post-hatching, n = 60 worms each) and 4 hatchling pools (< 1 week post-hatching, n = 300 worms each). Worms were starved for 24 hours, after which RNA was extracted using NucleoSpin™ RNA XS columns (Macherey-Nagel, Düren, Germany), separately for each pool. A subset of material from each replicate pool (1 µl, ~850ng RNA) was then used to make one combined mRNA sample for PacBio Iso-Seq library preparation, while the remaining replicate pools were used for Illumina cDNA library preparation. Gene expression contrasts in adult vs. hatchling worms have previously been made for the SR1 inbred line of *M. hystrix* (Brand et al. 2020) and for *M. lignano* (Wiberg et al. 2022). We obtained the raw-data associated with these studies from NCBI and ENA (table S1Ab). For *M. hystrix* a new set of 2 adult pools (n = 60 worms each) and 2 hatchling pools (n = 300 worms each) was produced as above to generate one combined mRNA sample for an Iso-Seq library (see table S1Ab).

Iso-Seq cDNA libraries were prepared using the protocol for "Iso-Seq™ Express Template Preparation for Sequel® and Sequel II Systems" (Pacific Biosciences, Menlo Park, CA, USA). Each library was sequenced on a single 1M SMRT cell of the PacBio Sequel platform at the GFB and subsequently on a single 8M SMRT cell of the PacBio Sequel II platform at the Lausanne Genomics Technologies Facility (University of Lausanne; for details see table S1Ab).

Illumina cDNA libraries were prepared at the GFB from >100ng (119 – 348ng) total RNA using the TruSeq Stranded mRNA Library Prep Kit High Throughput (Illumina, San Diego, CA, USA) and the TruSeq RNA CD Index Plate (Illumina) producing unique pairs of indexes for each sample. Following 15 cycles of PCR the libraries were multiplexed, and sequenced on one lane of the NovaSeq 6000 platform at the GFB for 101bp PE reads.

2.3 RNA extraction and sequencing: positional RNA-Seq datasets

Since no methods currently exist to dissect individual organs in *Macrostomum* we used a positional RNA-Seq approach to identify transcripts enriched in certain body regions containing organs and tissues of interest. The cutting levels follow the original logic presented in Arbore et al. (2015) but due to anatomical differences between species we adapted the placement of the cutting levels to ensure we compared similar body fragments (see figure 1C and D). Cutting level A was placed anterior to the testis in both *M. lignano* and *M. cliftonense,* and towards the anterior end of the pharynx in *M. hystrix* due to the more anterior position of the tip of the testis in that species (see figure 1C). Cutting level B was placed anterior to a distinct notch in the gut between the testes and ovaries to decrease the likelihood of finding ovary-specific RNA in the testis samples (see 'N' in figure 1C). Finally, cutting level C was placed posterior to the ovaries, ensuring enrichment of ovary RNA in this fragment containing the head, the testes, and the ovaries.

Throughout we use the following definitions when referring to cutting levels and the resulting samples (figure 1D): A 'region' is a segment of the worm between two adjacent cutting levels and a 'fragment' is the part of the worm anterior to a cutting level. Worms were thus compartmentalised into four distinct regions, separated by three cutting levels, that produced four fragments. The head region is situated anterior to cutting level A. Between cutting levels A and B lies the testis region, which principally contains the testes and part of the gut (figure 1C and D). Between cutting levels B and C is the ovary region, which contains the ovaries, another section of the gut, the growth zone posterior to the ovaries, and thus some signal from the developing eggs. Finally, the tail region, which contains the male stylet, seminal vesicle, developing eggs, and the female antrum (figure 1C and D).

To obtain worms of standardised age and condition for positional RNA-Seq, adult worms of unknown age were transferred to standard culture petri dishes (*M. lignano* N ≈ 600; *M. cliftonense*

N ≈ 480; *M. hystrix* N ≈ 720). Worms were allowed to mate and lay eggs for three days before transfer to fresh dishes, followed by a second three-day period. This resulted in two batches of hatchlings of standardised age per species (*M. lignano* N ≈ 500 x2; *M. cliftonense* N ≈ 400 x2; *M. hystrix* N ≈ 360 x2). These batches were then allowed to mature to adulthood, being transferred to fresh algae plates every 8-12 days. Due to differences in development time, the maturation period and number of transfers differed across species (days since end of laying period/no. of transfers: *M. lignano* = 30/3; *M. cliftonense* = 39/5; *M. hystrix* = 50/6). Worm maturity was determined by checking worms for the presence of male and female gonads and genitalia, mature eggs in the female antrum, and laid eggs in the culture dishes. Finally, batches were starved for 24 hours in a petri dish with 20 ml of ASW and no algae. This allows for better visibility of gonads during dissection and should reduce the representation of algal RNA in the final samples.

On each day of cutting, a master solution was prepared of 800 µl of Lysis Buffer RA1 and 16 µl TCEP reducing agent of the NucleoSpin™ RNA XS RNA isolation kit (Macherey-Nagel) and vortexed for several seconds. 35 µL of master solution was then transferred into 1.5 mL Eppendorf tubes that were stored in a refrigerator. Single worms were transferred onto a glass slide in 5 µl of ASW and cut using a scalpel under a dissecting microscope. To avoid any bias from worms with particular traits being chosen for particular cutting levels, the cutting level for each worm was only revealed, according to a previously prepared randomised order, once the worm had been selected for cutting. Fragments were then placed directly into lysis buffer, with as little ASW as possible. This process, from cutting to lysis, was performed as swiftly as possible, taking no more than 20 seconds. The scalpel and glass were rinsed with water and wiped dry between usage to prevent cross-contamination of samples. This process was repeated, to generate four biological replicates per fragment and per species. In *M. lignano*, two replicates could be produced in a single day, while in *M. cliftonense* and *M. hystrix* only a single replicate could be produced in a single day due to the larger total number of worms required (see figure 1C and D, and table S1Ac).

Each replicate was split into two sub-replicates, and total RNA extraction was then performed using NucleoSpin™ RNA XS columns (Macherey-Nagel) with two elution steps to recover as much RNA as possible, first using 10µl and then with 8.5µl RNA/DNA extraction buffer (Macherey-Nagel). Samples were stored at -80°C prior to library preparation and sequencing.

Illumina cDNA libraries were prepared at the GFB from 100ng total RNA, where possible (40/48 samples), using the TruSeq Stranded mRNA Library Prep Kit High Throughput (Illumina, San Diego, CA, USA) and the TruSeq RNA CD Index Plate (Illumina). The libraries were multiplexed, and sequenced on two lanes of the NovaSeq6000 platform at the GFB for 101bp SE reads (table S1Ac).

2.4 RNA extraction and sequencing: individual worm samples

We here produce novel sequencing data from individual *M. cliftonense*, *M. hystrix*, and *M. lignano*. Some RNA-Seq data from individual worms already exist from previous studies (Brand et al. 2020; Brand et al. 2022b; table S1Ad). We expanded this with wild-caught individual *M. cliftonense* and *M. hystrix* sampled from natural populations (figure S2) as described in Brand et al. (2020; 2022b) and stored in RNAlater (Life Technologies Corporation, Carlsbad, CA, USA), at -80°C, and with specimens of *M. lignano* sampled from the outbred LS3 laboratory culture (Zadesenets et al. 2016; Brand et al 2022b). Accession numbers, references, and additional details (sampling location and coordinates) for all samples are given in table S1Ad. RNA was extracted using NucleoSpin™ RNA XS columns (Macherey-Nagel), cDNA synthesis by SMART-Seq v4 low-input kits (Clontech Laboratories, Inc.), followed by Nextera XT (Illumina) library preparation. Sequencing was performed on NovaSeq6000 at the GFB (table S1Ad).

3. Genome assembly

Genome assembly and annotation proceeded in several steps, an overview of which is shown in figure 1A and B. In this and the next section the steps of assembly (figure 1A) and annotation (figure 1B) pipeline are described in detail. Sample code and software commands for the assembly and annotation pipelines, as well as differential expression analyses can be found in the associated zenodo repository (https://doi.org/10.5281/zenodo.7861770). Other tools used in the processing of data and assemblies were samtools (v. 1.10; Li et al., 2009) and BamTools (v. 2.5.1; Barnett et al., 2011).

3.1 Initial genome assembly – M. cliftonense

Raw PacBio reads were assembled with canu (v. 1.8; Koren et al. 2017) with default parameters. This assembly produced a much longer genome (~435Mb) than expected, at nearly double the size obtained from fluorescence-based genome size estimates (~230Mb; Schärer et al. 2020). In addition, we used BUSCO (v. 4.0.6; Simão et al. 2015) with the metazoa_odb10 set of conserved genes to assess completeness of the genome and found that while 84% of the genes were found as complete, 51.9% of these were present as duplicates. This points to residual heterozygosity within the inbred line used for sequencing, resulting in the assembly of redundant contigs representing different haplotypes. Similarly, k-mer copy number spectra comparisons from Merqury (Rhie et al. 2020) using the Illumina short-read data showed that a substantial number of k-mers were present in multiple copies in the assembly (figure 2A). Such patterns also indicate residual heterozygosity and multiple haplotypes. Multiple haplotypes (>2) are not expected from a diploid

genome, but the combination of residual heterozygosity, the use of a large pool of individuals (and therefore multiple recombinant individuals), and long-read sequencing, may result in multiple assembled haplotypes. We therefore used Purge Haplotigs (v. 1.1.1; Roach et al. 2018) to filter out putative heterozygous regions. This tool uses genome-wide coverage statistics to identify contigs that are likely homozygous vs. heterozygous. Heterozygous regions should have half the coverage of homozygous regions, because half the reads from such regions will be generated from, and thus map to, one or the other haplotype. Meanwhile, homozygous regions are not split into haplotypes by the assembler and thus produce a single sequence to which all reads from that region will map. The distribution of coverage should therefore look very distinctive with, in the extreme case, two modes. For *M. cliftonense*, the distribution of coverage throughout the genome produced a curve characteristic of excess heterozygosity (figure S3). There is an apparent "shoulder" at a coverage of ~50-60x (figure S3), suggesting underlying coverage distributions with means of ~25x (representing haplotigs) and ~60x (representing homozygous regions). We therefore set coverage thresholds at 5 (min), 55 (middle), and 190 (high) to delineate the coverage expected at contigs that are assembled from heterozygous (between 5x and 55x coverage) and homozygous (between 55x and 190x coverage) regions (figure S3). Varying the middle coverage threshold between 45 and 55 did not substantially influence the final assembly (data not shown).

3.2 Initial genome assembly – M. hystrix

Also here, raw PacBio reads were assembled with canu with default parameters. This assembly also produced a longer genome (290Mb) than expected from fluorescence-based genome size estimates (~217Mb; Schärer et al. 2020). BUSCO analysis found 84.7% of the genes as complete sequences, but with 26.2% present as duplicates, suggesting some residual heterozygosity also within the highly inbred SR1 line. Here too, k-mer copy number spectra comparisons from Merqury using the Illumina short-read data showed the presence of many multi-copy k-mers in the assembly, but the evidence only suggests a maximum of two haplotype assemblies (figure 2A) We therefore again used Purge Haplotigs to separate the assembled haplotypes. The distribution of coverage from PacBio raw reads throughout the genome produced a curve indicating excess heterozygosity with a more bi-modal distribution than seen in *M. cliftonense* (figure S3). For *M. hystrix* we set coverage thresholds at 2 (min), 22 (middle), and 190 (high), to delineate the coverage expected at contigs that are assembled from heterozygous and homozygous regions as above. These parameters produced a smaller genome assembly (220Mb) that is very similar to the above-mentioned genome size estimate. Moreover, BUSCO results indicated that while the proportion of complete genes identified did not drop substantially (from 84.7% to 84.5%) the proportion of complete BUSCO genes present

in multiple copies dropped from 26.2% to 6.2%. As above, varying the middle coverage threshold between 22 and 26 did not substantially influence the final assembly (data not shown).

3.3 Polishing of genome assemblies

Initial polishing of the genome assemblies was performed with Arrow/gcpp (v. 1.9.0) from the pb-assembly suite (available from https://github.com/PacificBiosciences/pbbioconda). This tool uses the raw PacBio reads, including quality information, to correct mis-assemblies and sequencing errors. We mapped raw PacBio reads with pbmm2 (v. 1.3.0; from the pb-assembly suite) to the assembly and performed four rounds of iterative polishing followed by quality assessment with QUAST (v. 5.0.2; Mikheenko et al., 2018) and BUSCO. Quality assessment shows that neither the N50 nor N75 increased substantially, suggesting that assemblies could not be made more contiguous (figure S4A; table S2A). Substantial increases in the number of predicted genes would have indicated that sequencing errors leading to frameshifts or premature stop codons (indels or single-site errors) were being corrected by polishing. However, the number of predicted genes was stable across iterations, with only minor improvements (figure S4A; table S2A). Similarly, although the number of complete BUSCOs identified did increase up to the second iteration in *M. hystrix*, this was not dramatic and indeed was worse than the raw assembly with purged haplotigs for *M. cliftonense* (table S2B). Nevertheless, the overall best values were achieved with two iterations of Arrow/gcpp polishing. We therefore used this genome assembly for downstream steps and relied more on polishing using short reads.

We next performed additional polishing with Illumina short reads (table S1Aa). Reads were first trimmed to remove any remaining adapter sequences with Trimmomatic (ILLUMINACLIP:TruSeq3-PE-2.fa:2:30:10; Bolger et al. 2014). Trimmed reads were then mapped to the genome assembly from the Arrow/gcpp polishing steps (see above) with bwa mem (0.7.17; Li 2013; available from: https://github.com/lh3/bwa). We then used pilon (v. 1.23; Walker et al., 2014) to polish the assembly, considering only reads with a mapping quality > 10. We performed 9 rounds of iterative polishing followed by quality assessment of the assemblies with QUAST and BUSCO. After 5 rounds of polishing the number of changes made (i.e. SNPs or InDels corrected), did not continue to improve for either species (figure S4B; table S2B). After the initial round of polishing with pilon, neither the N50 nor the N75 values changed and BUSCO scores did not improve at any stage (table S2B). We therefore kept the assemblies after 5 rounds of polishing as the final genome assemblies for all further analyses. Merqury k-mer spectra and BUSCO scores of the final assemblies are presented in the results of the main text (see also figure 2A).

3.5 Identification and masking of repeat regions

To identify repeats within the genomes of *M. cliftonense* and *M. hystrix* we first modelled repeats for each genome assembly with RepeatModeler (v. 2.0.1; Smit et al., 2015a), with the "-LTRStruct" option to identify long terminal repeats (LTRs). We then used RepeatMasker (v. 4.1.1; Smit et al., 2015b) to quantify the abundance of the identified repeats in each genome and to produce a "soft-masked" genome assembly. We also ran this procedure for the *M. lignano* genome (Mlig_3_7, available from: http://gb.macgenome.org/downloads/Mlig_3_7/) for comparison, because repeats were identified with a slightly different methodology in Wudarski et al., (2017).

4. Genome annotation

We made use of several lines of evidence in the annotation of the above genome assemblies. This consisted of Illumina RNA-Seq and PacBio Iso-Seq data, which were used to assemble transcripts and guide *ab intio* gene prediction. An overview of the pipeline is given in figure 1B. Below, we describe the processing and combination of each type of data in more detail.

4.1 Identification of spliced leader (SL) sequences

Trans-splicing of a spliced leader (SL) sequence has been observed in several flatworm species (Rajkovic et al. 1990; Davis et al. 1995; Davis 1997; Zayas et al., 2005; Lasda & Blumenthal 2011; Bitar et al., 2013), including members of the genus *Macrostomum* (Wasik et al. 2015; Grudniewska et al. 2018; Wudarski et al. 2017; Ustyantsev & Berezikov 2021). Although the precise function of SL *trans*-splicing in flatworms is still unknown, a common function of *trans*-splicing is to delineate and cap mature mRNA derived from polycistronic transcripts (Zayas et al., 2005; Lasda & Blumenthal 2011; Bitar et al., 2013). In addition, all known flatworm SL sequences terminate in AUG, which is sometimes also used as the translation initiation site (Lasda & Blumenthal 2011). Thus, identifying locations of SL incorporation can help with delineating gene boundaries (Wudarski et al., 2017). Moreover, accurate mapping of RNA-Seq reads to the genome assemblies and subsequent annotation and transcript assembly may be compromised by the inclusion of the spliced leader (SL) sequence in reads. We therefore aimed to first identify the SL sequence in the RNA-Seq reads, in order to remove this sequence prior to mapping and to also selectively map those reads that include SL sequence information. *De novo* transcriptomes for both *M. cliftonense* and *M. hystrix* have previously been assembled (Brand et al. 2020; Brand et al. 2022a;2022b; table S1Af). In addition, candidate SL sequences are available for *M. lignano* (Wasik et al., 2015; Grudniewska et al., 2016).

We took an approach similar to that described by previous authors (Wasik et al., 2015; Grudniewska et al., 2016) to identify the SL sequence from assembled transcripts of *M. cliftonense* and *M. hystrix*. We extracted the first 100nt of each assembled transcript from the available *de novo*

transcriptome assemblies. We then produced all possible 19-mers from these sub-sequences using jellyfish (v. 2.3.0; Marçais & Kingsford 2011) and retained all 19-mers present in copy numbers of > 1,000. We then assembled these 19-mers using velvet (v. 1.2.10; Zerbino & Birney 2008), using a hash size of 10nt (parameter settings: -cov_cutoff 0 -min_contig_lgth 10 -coverage_mask 0 -exp_cov 3). The resulting contigs were aligned to previously published SL sequences from *M. lignano* (Wasik et al. 2015; Grudniewska et al. 2016; figure 2C; table S3). Where these sequences overlapped in the alignment, they were identical across all three species. Our approach produced a single contig for *M. cliftonense*, which was one base longer than the *M. lignano* SL sequence of Grudniewska et al. (2016) and identical to that of Wasik et al. (2015) (table S3; figure 2C). Also, for *M. hystrix* a single sequence was produced, which was near identical to the other SL sequences, but with an extra cytosine at the 3' end, likely due to mis-assembly, since SL-sequences are expected to terminate with a translation start codon (Lasda & Blumenthal 2011; table S3; figure 2C). Mis-assembly at the 3' end is likely due to the fact that the initial 19-mers are sampled from a diverse set of mRNA fragments that all contain more than simply the SL-sequence at their 3' end. By chance, assembly algorithms may then find support for additional, spurious bases. The final sequences used for trimming of the RNA-Seq reads (see below) are boxed by dashed lines in figure 2C.

4.2 Illumina RNA-Seq data

We performed mild trimming of raw Illumina RNA-Seq reads with Cutadapt (v. 3.0; Martin 2011) to remove TruSeq adapters (-a AGATCGGAAGAGCACACGTCTGAACTCCAGTCA -A AGATCGGAAGAGCGTCGTGTAGGGAAAGAGTGT --minimum-length 50 --nextseq-trim=25). In a second round of trimming, the identified SL sequences (table S3) were used as a custom input clipping file, using the settings (-g [*SL-sequence*] -O 6 --minimum-length 50). RNA-Seq reads were then mapped with STAR (v. 2.7.3a; Dobin et al. 2013). For TruSeq Stranded libraries, sequences present at the 5' end of mRNAs (such as spliced SL sequences) should only be present on the R2 read. Thus, to identify reads that contain SL sequence we ran Cutadapt in single-end mode and with the "--info-file" option enabled on just the R2 reads. Reads from which the SL sequence was trimmed were then extracted and mapped separately with STAR. We then called *trans*-splicing locations by identifying regions with a coverage of these SL-containing reads > 10x or > 100x. Coverage thresholds are arbitrary, but a lower limit of coverage is desirable to avoid over-calling SL sites due to spurious matches. We note that the choice of threshold does not impact our conclusions regarding *trans*-splicing of polycistronic genes (see the results in the main text).

The mapped RNA-Seq reads were used as input to BRAKER (v. 2.1.5; Hoff et al., 2016; 2019), which first uses this data as input to GeneMark-ET (v. 4.62; Lomsadze et al., 2014) to train and

build initial gene models. Next, BRAKER provides a high-quality training set of the initial gene models as an input set for AUGUSTUS (v. 3.4.0; Stanke et al., 2006; 2008) to perform *ab intio* gene prediction, along with RNA-Seq information to inform on intron boundaries and untranslated regions (UTRs; --UTR=on). BRAKER was run using genome assemblies with soft-masked repeats.

4.3 PacBio Iso-Seq data

High-quality full-length transcripts were identified from Iso-Seq circular consensus sequence (CCS) reads using the IsoSeq3 pipeline (available from: https://github.com/PacificBiosciences/pbbioconda). These transcripts were then mapped to the genome assembly with minimap2 (v. 2.17; Li 2018) in splice-aware mode. Helper scripts from the cDNA_Cupcake package (v. 12.4.0; available from https://github.com/Magdoll/cDNA_Cupcake) were then used to further reduce redundancy of high-quality transcripts by clustering them based on genome mapping locations (collapse_isoforms_by_sam.py).

4.4 Data integration and detecting trans-spliced genes

Finally, we used PASA (v. 2.4.1; Haas et al., 2003; Campbell et al., 2006; Haas 2011) to integrate full-length transcript information, from the processed Iso-Seq data, with the gene models produced by BRAKER. We first used PASA to map Iso-Seq transcripts to the genome with both BLAT (v. 36x4; Kent 2002) and GMAP (v. 2017-11-15; Wu & Watanabe 2005) and created a database of mapping locations. These transcripts were then updated using the annotations produced by BRAKER. This integrated annotation was used as the final set of gene models. Basic statistics at the level of transcripts and genes for these annotations can be seen in table 2 and table S2C. Note that several annotated coding sequences are not "valid" (i.e. have lengths that are not multiples of three). This is a common occurrence in automated annotation pipelines and validation of such gene models will require additional investigation. These transcripts are typically shorter than other contigs, and may represent transcribed non-coding RNAs that nevertheless give rise to small open reading frames, making them difficult to annotate. Given the relatively low number of such "invalid" coding transcripts (*M. cliftonense*: N = 152 [0.2% of transcripts], *M. hystrix*: N = 26 [0.04% of transcripts]), we simply filter these out for all downstream analyses that rely on coding sequences.

To identify genes that are likely *trans*-spliced, we used the overlap subroutine of BEDtools (v. Quinlan & Hall 2010) to identify annotated genes that coincided with *trans*-splicing locations in the genome (as defined above). This gave an estimate of the proportion of genes that are *trans*-spliced. Wudarski et al., (2017) noted that ~7% of assembled transcripts were likely immature mRNA molecules representing multiple *trans*-spliced genes (polycistronic genes). We assessed whether and

how many of the assembled and annotated genes from the *M. cliftonense* and *M. hystrix* assemblies represented such immature mRNAs by checking whether an annotated gene contained multiple *trans*-splicing locations.

4.5 Ortholog identification

We identified ortholog groups (OGs) between *M. cliftonense*, *M. hystrix*, and *M. lignano* with Proteinortho (v. 6.0.29; Lechner et al., 2011) using the PoFF (Lechner et al., 2014) feature. We first filtered the annotated transcripts to include only the longest isoform for each gene. We then obtained the order of genes along each contig from the annotation.gff3 files for each species. Finally, we ran Proteinortho with the PoFF module (-synteny) to infer OGs using default parameter settings.

5. Differential gene expression analyses

5.1 Positional RNA-Seq

Reads from each replicate pool of worm fragments were concatenated across the two sequencing lanes. Upon manual inspection of the data, we observed several transcripts in *M. lignano* for which the first replicate of the Head and Testis fragments had very similar counts to all replicates of the whole-worm fragments. This was most obvious where the other Head and Testis fragments had counts of 0 across the remaining replicates. The most likely explanation is that these Head and Testis replicates were contaminated with at least one whole-worm fragment. Due to this apparent handling error, we thus excluded from the subsequent analyses the first replicates for the Head and Testis fragment pools from *M. lignano* (Maclig_Head1 and Maclig_Testis1 in table S1Ac), resulting in only three rather than four replicates for these fragments.

TruSeq sequencing adapters were trimmed using Trimmomatic (ILLUMINACLIP). We then used salmon (v. 1.8.0; Patro et al., 2017) to quantify expression levels for all samples. The *M. cliftonense* and *M. hystrix* samples were mapped to the newly annotated transcripts (n = 73,177 and n = 54,873 transcripts, respectively); while for *M. lignano* we used the Mlig_RNA_3_7_DV1.v3.coregenes transcriptome assembly (Grudniewska et al., 2018; n = 90,195 transcripts). We applied the salmon corrections for GC content and position specific biases. Salmon excludes exact duplicate transcripts, resulting in expression estimates for a total of 71,138, 53,978, and 87,881 transcripts for *M. cliftonense*, *M. hystrix,* and *M. lignano*, respectively. We performed minimal filtering of transcripts to exclude those with zero read counts across all samples (*M. cliftonense*, n = 16,695; *M. hystrix*, n = 8,679; *M. lignano*, n = 8,323). In addition, we removed transcripts with very low expression (overall counts across all samples < 16; *M. cliftonense*, n =

4,619; *M. hystrix*, n = 3,579; *M. lignano*, n = 5,856). The final remaining number of transcripts were 49,824, 41,720, and 73,702 for *M. cliftonense*, *M. hystrix*, and *M. lignano*, respectively.

We performed simple PCA using the 'prcomp()' function of the 'stats' package (), with all transcripts as input, to explore overall expression patterns across fragment samples. We next used DESeq2 (v. 1.30.1; Love et al., 2014) to test for pairwise differences in expression between adjacent worm fragments. In total, three separate fragment contrasts were performed: testis fragment vs. head fragment (T v H); ovary fragment vs. testis fragment (O v T); and whole-worm vs. ovary fragment (W v O). Transcripts were then classified according to the results of these three separate contrasts, based on which the annotation category for each transcript was derived (table S4). These annotation categories correspond to the same logic of expression patterns across increasingly larger worm fragments outlined in Arbore et al. (2015). Note that the final category, labelled "non-specific" throughout this study, has previously been labelled "non-differentially expressed" (Arbore et al. 2015), "non-specific" (Brand et al. 2020), or "ubiquitously-expressed" (Wiberg et al. 2021;2022). Final expression data, individual contrast results, and annotations for each species are given in table S5 and summarised in table S6. Although we categorise transcripts into two distinct ovary annotation groups, throughout our results we combine the two into a single ovary category, except where otherwise noted.

For *M. lignano*, we additionally took advantage of previously generated *in situ* hybridisation (ISH) data to validate annotated expression patterns of a subset of transcripts. Specifically, Weber et al. (2018) and Lengerer et al. (2018) aimed to confirm the expression patterns of all transcripts annotated as tail-region specific in Arbore et al. (2015). Detailed descriptions of expression patterns, along with ISH probe information, are available for 310 out of 366 tail-region annotated transcripts (table S8). Using blastn, we first associated transcript sequences from the current transcriptome assembly to sequences (and IDs) from a previous transcriptome assembly, from which these ISH probes had originally been designed (MLRNA110815; Simanov 2014; available from https://www.macgenome.org/download/MLRNA110815/). The top hit for each MLRNA110815 transcript was taken (ranked first by e-value and any ties broken by bit-score). In this way, we could assign new transcript IDs to 308 of the 310 tail-region transcripts with ISH expression patterns. From the detailed descriptions of ISH expression patterns, we then identified those that could be described as tail-region specific, i.e. those which indicated expression in tissues restricted to the tail-region of the worm (see figure 1C and D). We then compared these annotations against the positional annotation obtained from the bioinformatic analysis of the positional RNA-Seq data. We tested whether the new positional RNA-Seq data established for *M. lignano* in the current study was more successful at identifying tail-region specific transcripts than the previous data and analyses that lacked statistical replication (Arbore et al. 2015; Brand et al. 2020). Specifically, we computed

the proportion of transcripts with tail-region annotations from the bioinformatic analysis of RNA-Seq data that were also identified as tail-region specific from the results of ISH experiments.

Using the inferred orthology relationships among genes (see above) we identified OGs with shared annotations across the three species. Ambiguity in annotation and ortholog relationships might arise due to mis-assembly or mis-annotation of genes, especially in *M. lignano* where a whole-genome duplication has occurred (Zadesenets et al. 2017a; 2017b). We therefore define three sets of OGs with conserved annotations. The strictest set of OGs identified was those with a single representative sequence from each species (one-to-one OGs), where all sequences had the same annotation (called the strict set). In addition to these, we also identify those OGs with the same number of sequences from each species, and where all sequences had the same annotation (called the relaxed set). Finally, we also identified OGs with a variable number of sequences from each species (but with a minimum of one) where all sequences had the same annotation (called the liberal set). We exclude genes with no annotation and those in the "other" annotation category.

5.2 Adult vs. hatchling RNA-Seq data analysis

A contrast of gene expression in adult vs. hatchling worms using the most recent transcriptome assembly is already available for *M. lignano* (Wiberg et al. 2022). Such a contrast should aid in identifying truly reproduction-related genes because such genes are generally not expected to be expressed in non-reproductive juveniles. RNA-Seq data from adults and hatchlings from *M. cliftonense* (this study; table S1Ab) and *M. hystrix* (from Brand et al. 2020; table S1Ab) were used to quantify expression of transcripts of the newly annotated genomes described above (as in Wiberg et al. 2022). Briefly, we trimmed remaining TruSeq adapter sequences using Cutadapt and then quantified expression of all transcripts in the respective transcriptome assemblies using the quasi-mapping approach of salmon. We performed minimal filtering to exclude transcripts with zero read counts in all samples, as well as all transcripts with read counts < 10 across all samples. Finally, we tested for differential expression in adult vs. hatchling worms with DESeq2. We called transcripts as differentially expressed if they had an FDR corrected p-value < 0.05. We identified all transcripts that were differentially expressed and had higher expression in adults compared to hatchlings (annotated as "Up-in-A") or higher expression in hatchlings compared to adults (annotated as "Up-in-H"). Final expression data, individual contrast results, and annotations for each species are given in table S9.

6. Assembly and annotation of the mitochondrial genome

To annotate the mitochondrial genomes of the two study species, we used published partial *cytochrome c oxidase I* (COI) sequences for *M. cliftonense* and *M. hystrix* (Janssen et al., 2015;

Schärer et al., 2020; see table S1Ae) to identify contigs in the assembled genomes that could represent the mitochondrial genomes. Specifically, using blastn (v. 2.2.29; Altschul et al., 1990; Camacho et al. 2009) we could confirm that the *M. cliftonense* assembly included a single contig, namely tig00002844, with good hits to these known sequences (all with identity >99% and e-value = 0.0). This contig was also labelled as circular by canu in the initial assembly, suggesting that this was the assembled mitochondrial genome. This contig was supported by only a few corrected long reads (n = 34), likely as a result of the combination of size selection of gDNA fragments >20kb and the read correction steps. For *M. hystrix*, neither of the two partial COI sequences yielded a hit in the final genome assembly. Given the combination of lower coverage of the PacBio data for *M. hystrix,* the fragment size selection applied, the initial read correction step of canu, and the final mitochondrial genome size of ~20kb (see results), the most likely explanation is that the mitochondrial genome was not adequately represented among the final assembled reads of the PacBio library, and thus simply did not assemble. We therefore removed the identified contig from the *M. cliftonense* assembly and took additional steps to independently assemble the mitochondrial genomes for both species from the Illumina short read data using the software MITObim (v. 1.9.1; Hahn, et al. 2013).

We used the published mitochondrial genome of *M. lignano* (Egger et al., 2017; Wudarski et al., 2017) as the initial bait sequence to catch reads mapping to highly conserved regions. These reads were then initially assembled into contigs that were subsequently used to catch more reads. Iterative baiting and assembly then extended the assembly from the conserved regions (Hahn et al. 2013). We assembled contigs of ~15.8kb and ~20.5kb in *M. cliftonense* and *M. hystrix*, respectively, which are both longer than the published sequence for *M. lignano* (~14.2kb). We confirmed the identity of these contigs based on good blastn hits of the known COI sequences from each species (all with identity > 99% and e-value < 0.001). Additionally, we performed further annotation of the mitochondrial genome with the MITOS2 webserver (table S2E), which does not rely on RNA-Seq expression data (beta version; Bernt et al., 2013).

Literature cited

Altschul, A.F., Gish, W., Miller, W., Myers, E.W. & Lipman, D.J. (1990). A basic local alignment search tool. *Journal of Molecular Biology.* **215**: 403-410.

Andersen, R.A., Berges, J.A., Harrison, P. J. & Watanabe, M.M. (2005). Recipes for freshwater and seawater media, in *Algal Culturing Techniques* (R.A. Andersen, ed.), Pp. 429-538. Elsevier, Amsterdam.

Arbore R. Sekii, K., Beisel, C., Ladurner, P., Berezikov, E. & Schärer, L. (2015). Positional RNA-Seq identifies candidate genes for phenotypic engineering of sexual traits. *Frontiers in Zoology.* **12**: 14.

Barnett, D.W., Garrison, E.K., Quinlan, AR., Strömberg, M.P. & Marth, G.T. (2011). Bamtools: A C++ API and toolkit for analyzing and managing BAM files. *Bioinformatics*. **27**: 1691-1692.

Bernt, M., Donath, A., Jühling, F., Externbrink, F., Florentz, C., Fritzsch, G., Pütz, J., Middendorf, M. & Stadler, P.F. (2013). MITOS: Improved de novo metazoan mitochondrial genome annotation. *Molecular Phylogenetics and Evolution*. **69**: 313-319.

Bitar, M., Boroni, M., Macedo, A.M., Machado, C.R. & Franco, G.R. (2013). The spliced leader trans-splicing mechanism in different organisms: molecular details and possible biological roles. *Frontiers in Genetics*. **4**: 199.

Bolger, A.M., Lohse, M. & Usadel, B. (2014). Trimmomatic: A flexible trimmer for Illumina sequence data. *Bioinformatics*. **30**: 2114-2120.

Brand, J.N., Wiberg, R.A.W., Pjeta, R., Bertemes, P., Beisel, C., Ladurner, P. & Schärer, L. (2020). RNA-Seq of three free-living flatworm species suggests rapid evolution of reproduction-related genes. *BMC Genomics*. **21**: 462.

Brand, J.N., Harmon, L.J. & Schärer, L. (2022a). Frequent origins of traumatic insemination involve convergent shifts in sperm and genital morphology. *Evolution Letters.* **6**: 63-82.

Brand, J.N., Viktorin, G., Wiberg, R.A.W., Beisel, C. & Schärer, L. (2022b). Large-scale phylogenomics of the genus *Macrostomum* (Platyhelminthes) reveals cryptic diversity and novel sexual traits. *Molecular Phylogenetics and Evolution*. **166**: 107296.

Camacho, C., Coulouris, G., Avagyan, V., Ma, N,. Papadopoulos, J., Bealer, K. & Madden, T.L. (2009). BLAST+: architecture and applications. *BMC Bioinformatics*. **10**: 421.

Campbell, M.A., Haas, B.J., Hamilton, J.P., Mount, S.M. & Buell, C.R. (2006). Comprehensive analysis of alternative splicing in rice and comparative analyses with *Arabidopsis*. *BMC Genomics.* **7**: 327.

Davis, R.E. Hardwick, C., Tavernier, P., Hodgson, S. & Singh, H. (1995). RNA Trans-splicing in flatworms. *Journal of Biological Chemistry*. **270**: 21813-21819.

Davis, R.E. (1997). Surprising diversity of spliced leader RNAs in flatworms. *Molecular and Biochemical Parasitology*. **87**: 29-48.

Dobin, A., Davis, C.A., Schlesinger, F., Drenkow, J., Zaleski, C., Jha, S., Batut, P., Chaisson, M. & Gingeras, T.R. (2013). STAR: ultrafast universal RNA-seq aligner. *Bioinformatics.* **29**: 15-21.

Egger, B., Bachmann, L. & Fromm, B. (2017). Atp8 is in the ground pattern of flatworm mitochondrial genomes. *BMC Genomics*. **18**: 414.

Grohme, M.A., Villa-Faré, M. & Rink, J.C. (2018). Small- and large-scale high molecular weight genomic DNA extraction from Planarians. in *Planarian Regeneration* (J. Rink ed.). Pp 267-275 Springer Science and Business Media, New York, NY.

Grudniewska, M., Mouton, S., Simanov, D., Beltman, F., Grelling, M., de Mulder, K., Arindrarto, W., Weissert, P.M., van der Elst, S. & Berezikov, E. (2016). Transcriptional signatures of somatic neoblasts and germline cells in Macrostomum lignano. *eLife*. **5**: e20607.

Grudniewska, M., Mouton, S., Grelling, M., Wolters, A.H.G., Kuipers, J., Giepmans, B.N.G. & Berezikov, E. (2018). A novel flatworm-specific gene implicated in reproduction in *Macrostomum lignano*. *Scientific Reports*. **8**: 3192.

Haas, B.J., Delcher, A.L., Mount, S.M., Wortman, J.R. Smith Jr, R.K., Hannick, L.I., Maiti, R., Ronning, C.M., Rusch, D.B., Town, C.D. Salzberg, S.L. & White, O. (2003). Improving the *Arabidopsis* genome annotation using maximal transcript alignment assemblies. *Nucleic Acids Research*. **31**: 5654-5666.

Haas, B.J. (2011). Analysis of alternative splicing in plants with bioinformatic tools. Pp 18-34 in *Nuclear pre-mRNA in Plants.* ASN Reddy & M Golovkin eds. Springer-Verlag. Berlin Heidelberg.

Hahn, C., Bachmann, L. & Chevreux, B. (2013). Reconstructing mitochondrial genomes directly from genomic next-generation sequencing reads - a baiting and iterative mapping approach. *Nucleic Acids Research* **41**: e129.

Hartfield, M. (2016). Evolutionary genetic consequences of facultative sex and outcrossing. *Journal of Evolutionary Biology.* **29**: 5-22.

Hoff, K.J., Lange, S., Lomsadze, A., Borodovsky, M. & Stanke, M. (2016). BRAKER1: Unsupervised RNA-Seq-based genome annotation with GeneMark-ET and AUGUSTUS. *Bioinformatics*. **32**: 767-769.

Hoff, K.J., Lomsadze, A., Borodovsky, M. & Stanke, M. (2019). Whole-genome annotation with BRAKER, in *Gene Prediction: Methods and Protocols* (M. Kollmar ed.). Pp 65-95. Springer Science and Business Media, New York, NY.

Janssen, T., Vizoso, D.B., Schulte, G., Littlewood, D.T.J., Waeschenbach, A. & Schärer, L. (2015). The first multi-gene phylogeny of the Macrostomorpha sheds light on the evolution of sexual and asexual reproduction in basal Platyhelminthes. *Molecular Phylogenetics and Evolution*. **92**: 82-107.

Kent, J.M. (2002). BLAT – The BLAST-like alignment tool. *Genome Research.* **12**: 656-664.

Koren, S. Walenz, B.P., Berlin, K., Miller, J.R., Bergman, N.H. & Phillippy, A.M. (2017). Canu: scalable and accurate long-read assembly via adaptive k-mer weighting and repeat separation. *Genome Research*. **27**: 722-736.

Lengerer, B., Wunderer, J., Pjeta, R., Carta, G., Kao, D., Aboobaker, A., Beisel, C., Berezikov, E., Salvenmoser, W. & Ladurner, P. (2018). Organ specific gene expression in the regenerating tail of *Macrostomum lignano*. *Developmental Biology*. **433**: 448-460.

Lasda, E.L. & Blumenthal, T. (2011). *Trans*-splicing. *Wiley Interdisciplinary Reviews: RNA*. **2**: 417-434.

Lechner, M., Findeiß, S., Steiner, L., Marz, M., Stadler, P.F. & Prohaska, S.J. (2011) Proteinortho: Detection of (Co-)orthologs in large-scale analysis. *BMC Bioinformatics* **12**: 124.

Lechner, M., Henandez-Rosales, M., Doerr, D., Wieske, N., Thévenin, A., Stoye, J., Hartmann, R.K., Prohaska, S.J., Stadler, P.F. (2014). Orthology detection combining clustering and synteny for very large datasets. *PLoS ONE*. **9**: e105015.

Li, H., Handsaker, B., Wysoker, A., Fennell, T., Ruan, J., Homer, N., Marth, G., Abecasis, G., Durbin, R. & The 1000 Genomes Project Data Processing Subgroup. (2009). The Sequence Alignment/Map format and SAMtools. *Bioinformatics*. **25**: 2078-2079.

Li, H. (2013) Aligning sequence reads, clone sequences and assembly contigs with BWA-MEM. arXiv:1303.3997v2.

Li, H. (2018). Minimap2: pairwise alignment for nucleotide sequences. *Bioinformatics*. **34**: 3094-3100.

Lomsadze, A., Paul, D.B., & Mark, B. (2014). Integration of mapped RNA-seq reads into automatic training of eukaryotic gene finding algorithm. *Nucleic Acids Research.* **42**: e119.

Love, M.I., Huber, W. & Anders, S. (2014). Moderated estimation of fold change and dispersion for RNA-seq data with DESeq2. *Genome Biology*. **15**: 550.

Martin, M. (2011). Cutadapt removes adapter sequences from high-throughput sequencing reads. *EMBnet Journal*. **17**: 10-12.

Marçais, G. & Kingsford, C. (2011). A fast, lock-free approach for efficient parallel counting of occurrences of k-mers. *Bioinformatic.* **27**: 764-770.

Mikheenko, A., Prjibelski, A., Saveliev, V., Antipov, D. & Gurevich, A. (2018). Versatile genome assembly evaluation with QUAST-LG. *Bioinformatics.* **34**: i142-i150.

Patro, R., Duggal, G., Love, M.I., Irizarry, R.A. & Kingsford, C. (2017). Salmon provides fast and bias-aware quantification of transcript expression. *Nature Methods*. **14**: 417-419.

Quinlan, A.R. & Hall I.M. (2010). BEDTools: a flexible suite of utilities for comparing genomic features. *Bioinformatics*. **26**: 841-842.

Rajkovic, A., Davis, R.E., Simonsent, J.N. & Rottman, F.M. (1990). A spliced leader is present on a subset of mRNAs from the human parasite *Schistosoma mansoni*. *Proceedings of the National Academy of Sciences of the United States of America.* **87**: 8879-8883.

Rhie, A., Walenz, B.P., Koren, S. & Phillippy, A.M. (2020). Merqury: Reference-free quality, completeness, and phasing assessment for genome assemblies. *Genome Biology*. **21**: 245.

Roach, M.J., Schmidt, S.A. & Borneman, A.R. (2018). Purge Haplotigs: allelic contig reassignment for third-gen diploid genome assemblies. *BMC Bioinformatics*. **19**: 460.

Schärer, L., Brand, J.N., Singh, P., Zadesenets, K.S., Stelzer, C.P. & Viktorin, G. (2020). A phylogenetically informed search for an alternative Macrostomum model species, with notes on taxonomy, mating behavior, karyology, and genome size. *Journal of Zoological Systematics and Evolutionary Research.* **58**: 41-65.

Simão F.A. Waterhouse, R.M., Ioannidis, P., Kriventseva, E.V. & Zdobnov, E.M. (2015). BUSCO: assessing genome assembly and annotation completeness with single-copy orthologs. *Bioinformatics*. **31**: 3210-3212.

Simanov, D. (2014). Genomic resources for the flatworm model organism *Macrostomum lignano*. Utrecht University: PhD thesis.

Smit, A.F.A., Hubley, R. (2015a). *RepeatModeler Open-1.0*. Available from: http://www.repeatmasker.org

Smit., A.F.A., Hubley, R. & Green, P. (2015b). *RepeatMasker Open-4.0.* Available from: http://www.repeatmasker.org

Stanke, M., Diekhans, M., Baertsch, R., & Haussler, D. (2008). Using native and syntenically mapped cDNA alignments to improve de novo gene finding. *Bioinformatics*. **24**: 637-644.

Stanke, M., Schöffmann, O., Morgenstern, B., & Waack, S. (2006). Gene prediction in eukaryotes with a generalized hidden Markov model that uses hints from external sources. *BMC Bioinformatics*. **7**: 62.

Ustyantsev, K.V. & Berezikov E.V. (2021). Computational analysis of spliced leader trans-splicing in the regenerative flatworm *Macrostomum lignano* reveals its prevalence in conserved and stem cell related genes. *Vavilov Journal of Genetics and Breeding*. **25**: 101-107.

Walker, B.J., Abeel, T., Shea, T., Priest, M., Abouelliel, A., Sakthikumar, S., Cuomo, C.A., Zeng, Q., Wortman, J., Young, S.K. & Earl, A.M. (2014). Pilon: an integrated tool for comprehensive microbial variant detection and genome assembly improvement. *PLoS ONE* **9**: e112963.

Wasik, K., Gurtowski, J., Zhou, X., Ramos, O.M., Delás, M.J., Battistoni, G., El Demerdash, O., Falciatori, I., Vizoso, D.B., Smith, A.D., Ladurner, P., Schärer, L., McCombie, W. R., Hannon, G.J. & Schatz, M. (2015). Genome and transcriptome of the regeneration-competent flatworm, *Macrostomum lignano*. *Proceedings of the National Academy of Sciences of the United States of America.* **112**: 12462-12467.

Wiberg, R.A.W., Viktorin, G. & Schärer, L. (2022). Mating strategy predicts gene presence/absence patterns in a genus of simultaneously hermaphroditic flatworms. *Evolution*. **76**: 3054-3066.

Wu, T.D. & Watanabe, C.K. (2005). GMAP: a genomic mapping and alignment program for mRNA and EST sequences. *Bioinformatics*. **21**: 1859-1875.

Wudarski, J., Simanov, D., Ustyantsev, K., de Mulder, K., Grelling, M., Grudniewska, M., Beltman, F., Glazenburg, L., Demircan, T., Wunderer, J., Qi, W., Vizoso, D.B., Weissert, P.M., Olivieri, D., Mouton, S., Guryev, V., Aboobaker, A., Schärer, L., Ladurner, P. & Berezikov, E. (2017). Efficient transgenesis and annotated genome sequence of the regenerative flatworm model *Macrostomum lignano*. *Nature Communications*. **8**: 2120.

Zadesenets, K.S., Schärer, L. & Rubtsov, N.B. (2017a). New insights into the karyotype evolution of the free-living flatworm *Macrostomum lignano* (Platyhelminthes, Turbellaria). *Scientific Reports*. **7**: 6066.

Zadesenets, K.S., Ershov, N., Berezikov, E. & Rubtsov, N. (2017b). Chromosome evolution in the free-living flatworms: first evidence of intrachromosomal rearrangements in karyotype evolution of *Macrostomum lignano* (Platyhelminthes, Macrostomida). *Genes*. **8**: 298.

Zayas, R.M., Bold, T.D. & Newmark, P.A. (2005). Spliced-leader *trans*-splicing in freshwater planarians. *Molecular Biology and Evolution*. **22**: 2048-2054.

Zerbino, D.R. & Birney, E. (2008). Velvet: Algorithms for de novo short read assembly using de Bruijn graphs. *Genome Research*. **18**: 821-829.

Supplementary tables and captions

Table S1. Sequencing datasets used in this manuscript, including NCBI BioProject and SRA accession numbers where applicable (see excel file: TableS1_sequencing_data.xlsx)

Table S2. Detailed genome assembly statistics (see excel file: TableS2_genome_assembly_stats.xlsx)

Table S3. Spliced leader (SL) sequence identified from the analysis of *de novo* transcriptome assemblies. The included start codon is highlighted in yellow. An alignment of the assembled sequences with the SL sequences previously identified in *M. lignano* is shown in figure 2C.

**Species**	**Assembled sequence**	**Length (nt)**
M. cliftonense	5’-GCCGTAAAGACGGTCTCTTACTGCGAAGACTCAATTTATTGC**ATG**-3’	45
M. hystrix	5’-CGTAAAGACGGTCTCTTACTGCGAAGACTCAATTTATTGC**ATG**-3’	43

Table S4. Positional RNA-Seq patterns yielding different annotation categories of reproduction-related transcripts. The table shows the required results from each of three fragment contrasts to call the final pattern indicative of a annotation category. All other combinations of results from the three contrasts is placed in an "Other" annotation category. FC – fold-change in expression.

**Annotation category**	**Required fragment contrast results**			**Final pattern**
	**T v H**	**O v T**	**W v O**	
Testis-region	$p_{adj} < 0.05$ AND $log_2FC > 0$	$p_{adj} > 0.05$	$p_{adj} > 0.05$	+, 0, 0
Ovary-region 1	$p_{adj} > 0.05$	$p_{adj} < 0.05$ AND $log_2FC > 0$	$p_{adj} > 0.05$	0, +, 0<br>+, +, 0
Ovary-region 2	$p_{adj} < 0.05$ AND $log_2FC > 0$	$p_{adj} < 0.05$ AND $log_2FC > 0$	$p_{adj} > 0.05$	
Tail-region	$p_{adj} > 0.05$	$p_{adj} > 0.05$	$p_{adj} < 0.05$ AND $log_2FC > 0$	0, 0, +
Non-specific	$p_{adj} > 0.05$	$p_{adj} > 0.05$	$p_{adj} > 0.05$	0, 0, 0

Table S5. Positional RNA-Seq results (see excel file: Table S5_positional_RNASeq_results.xslsx)

Table S6. The number (and percentage) of transcripts in each annotation category for each species.

	**Testis**	**Ovary**	**Tail**	**Non-Specific**	**Other**
M. cliftonense	5,598 (11%)	2,295 (5%)	307 (0.6%)	35,375 (71%)	6,249 (13%)
M. hystrix	7,383 (18%)	2,612 (6%)	204 (0.5%)	21,809 (52%)	9,712 (23%)
M. lignano	6,660 (9%)	646 (1%)	724 (1%)	62,454 (85%)	3,218 (4%)

Table S7. The agreement between annotations obtained for transcripts in this study and that of Brand et al. (2020). Numbers in the diagonal cells (in green) give transcripts that have the same annotation in both studies.

		**Brand et al. 2020**					
		**Testis**	**Ovary 1**	**Ovary 2**	**Tail**	**Non-specific**	**Other**
**This study**	**Testis**	2,649	37	10	14	2,144	139
	**Ovary 1**	12	61	30	2	184	12
	**Ovary 2**	19	46	48	0	77	1
	**Tail**	3	0	0	195	321	45
	**Non-Specific**	977	371	67	473	37,501	4,628
	**Other**	49	38	26	24	2,033	173

Table S8. Comparison of bioinformatic annotations to *in situ* hybridisation (ISH) experiment results. Shown are forward and reverse primer sequences that were designed for each transcript of a previous transcriptome assembly. Also given is a shorthand description of tissues or organs in which expression was observed from ISH experiments. Those transcripts with descriptions of expression patterns that could be described as Tail-region specific are labelled in the “ann_ISH” column. Also given is the best blast match from the new transcriptome assembly along with the annotation of this match in Brand et al. 2022, and in the current study (final column; see excel file: TableS8_annotations_to_ISH_comparison.xlsx).

Table S9. Adult vs. hatchling gene expression contrast results (see excel file: Table S9_AvH_RNAseq_results.xlsx). This table also contains the data for *M. lignano* as originally published in Wiberg et al. (2022).

Table S10. The number of annotated OGs for each of the positional annotation categories for three sets of OGs. The “strict” set includes all OGs with a single sequence from each species where all sequences have the same annotation. The “relaxed” set includes all OGs with the same number of sequences (≥ 1) from each species where all sequences have the same annotation (this set includes all OGs in the strict set). Finally, the “liberal” set includes all OGs with at least one sequence from each species where all sequences have the same annotation (this set includes both the strict and relaxed sets).

	**strict**	**relaxed**	**liberal**
**Testis**	383	385	578
**Ovary**	33	33	48
**Tail**	8	8	13
**Non-specific**	2,407	2,408	3,584

Table S11. The overlap between orthogroups (OGs) with consistent positional annotations and consistent patterns of differential expression in adult vs. hatchling worms, across species. Numbers include only OGs with a single representative from each species (one-to-one orthologs, n = 9,479).

	**Testis**	**Ovary**	**Tail**	**Non-specific**
**Not-DE**	3	0	1	804
**Up-in-A**	308	24	3	34
**Up-in-H**	0	0	0	39

Supplementary Figures and Figure Legends.

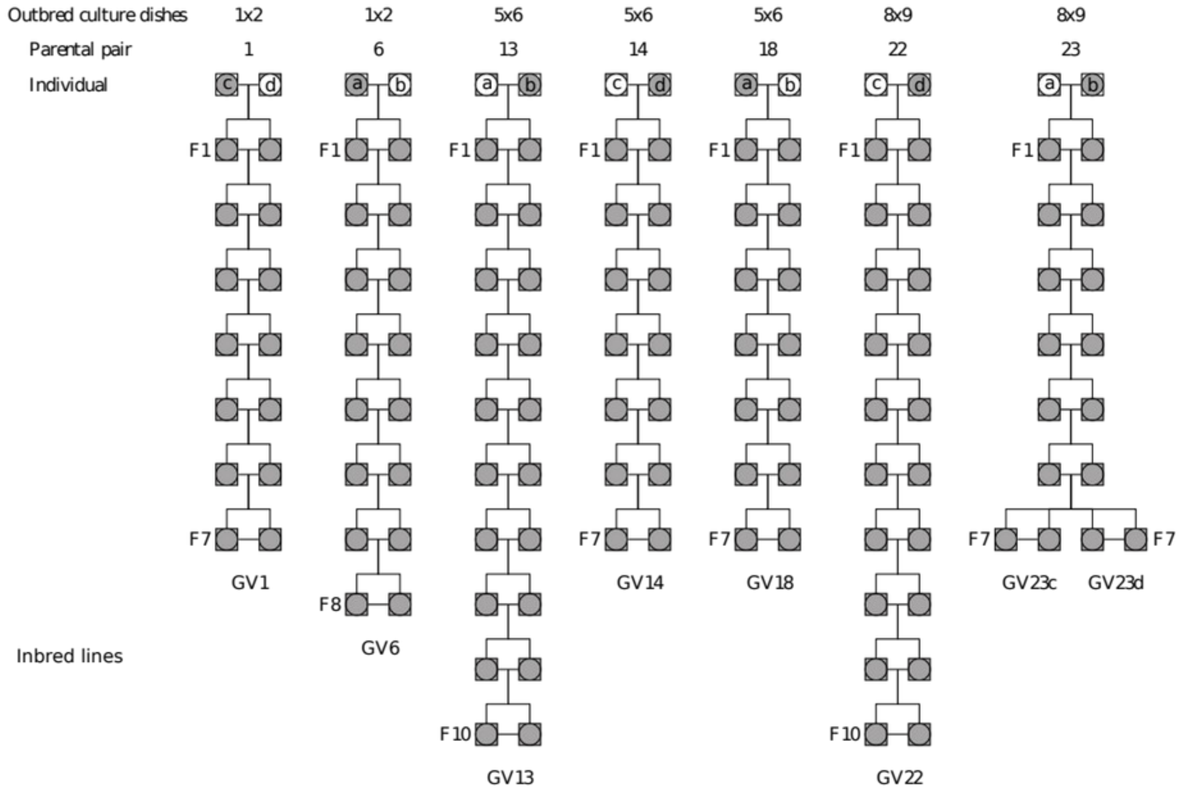

Figure S1. Diagram of the inbreeding procedure for *M. cliftonense.* Shapes filled in dark grey indicate individuals that were taken for the next generation. In the initial generation, only offspring from eggs laid by the grey individual of each pair were taken to the next generation. Note that although all final inbred lines are from independent initial pairs (except GV23c and GV23d), some of the pairs represent combinations of the same two outbred culture dishes (e.g., pairs 13, 14, and 18 are all combinations of dishes 5 and 6).

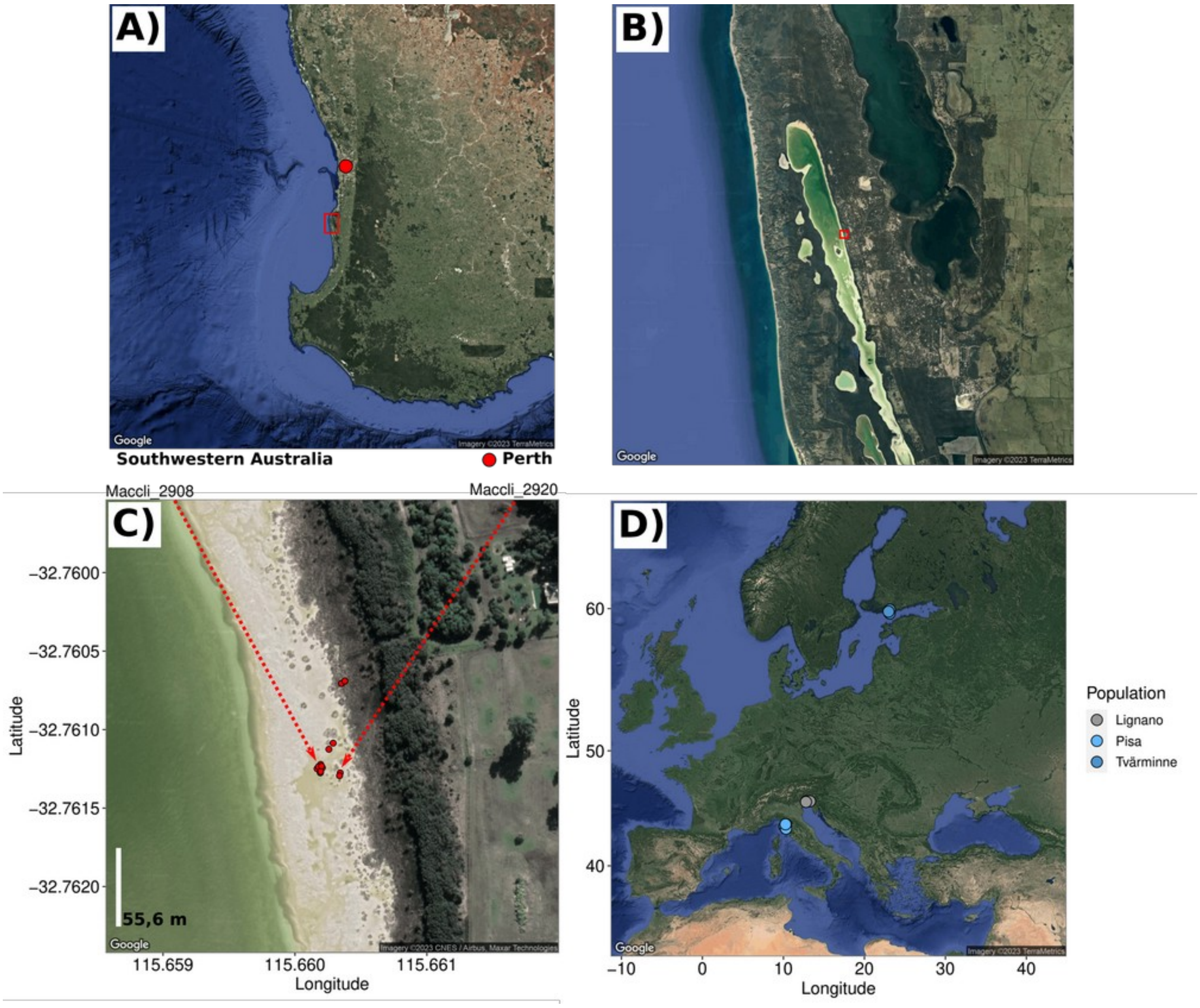

Figure S2. Sampling locations of wild-caught *M. cliftonense* and *M. hystrix* samples. Panels **A)**, **B),** and **C)**, give successively smaller scale views of the eastern shore of Lake Clifton, south of Perth, Australia, from which *M. cliftonense* samples originate. **D)** source populations of *M. hystrix* samples.

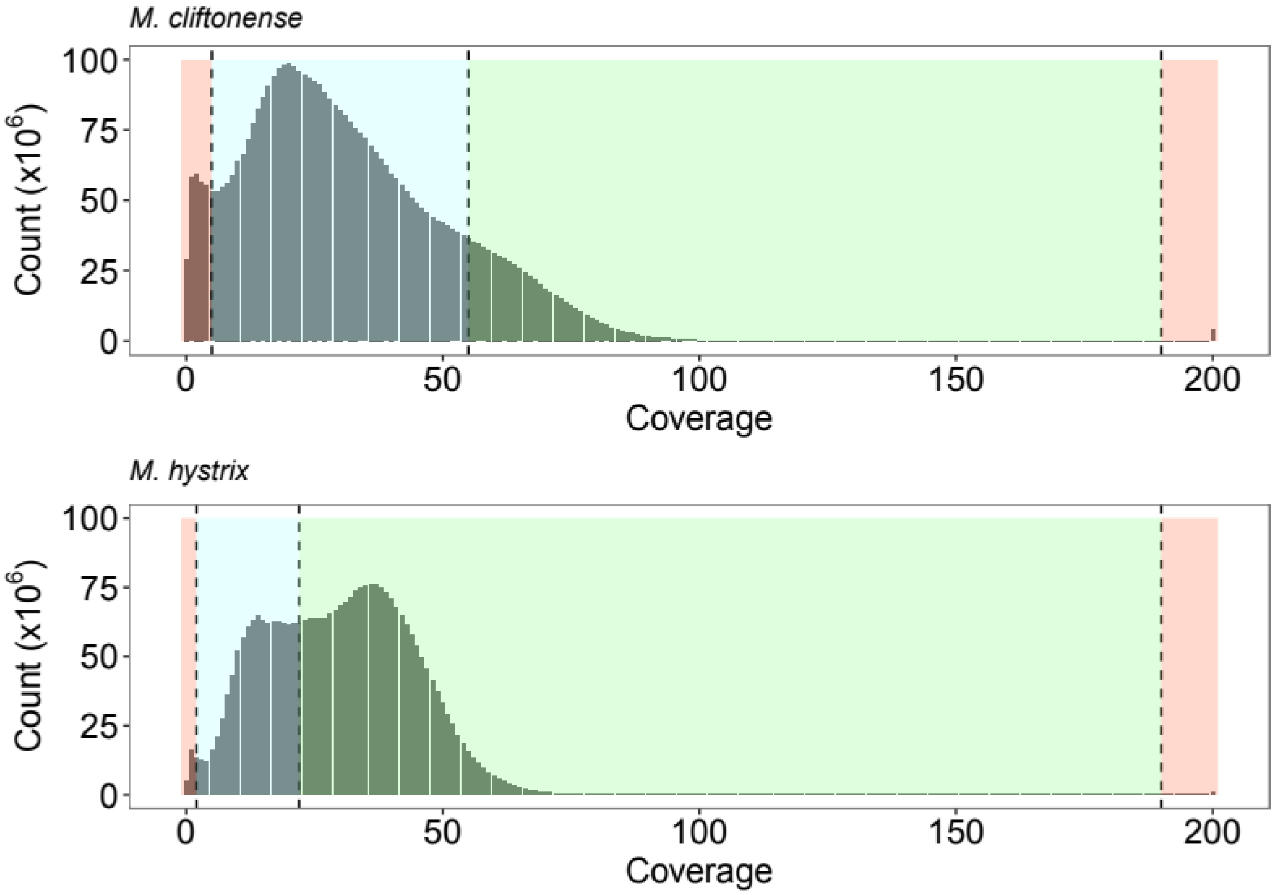

Figure S3. Coverage distribution across contigs from the initial assembly. Contigs from regions of the distributions in pink, are excluded due to very low or very high coverage. Contigs in the blue regions represent contigs with ~0.5x coverage relative to the rest of the genome, potentially indicating assembled haplotigs. Contigs in the green regions represent contigs from homozygous genomic regions that were correctly assembled as a single haplotype.

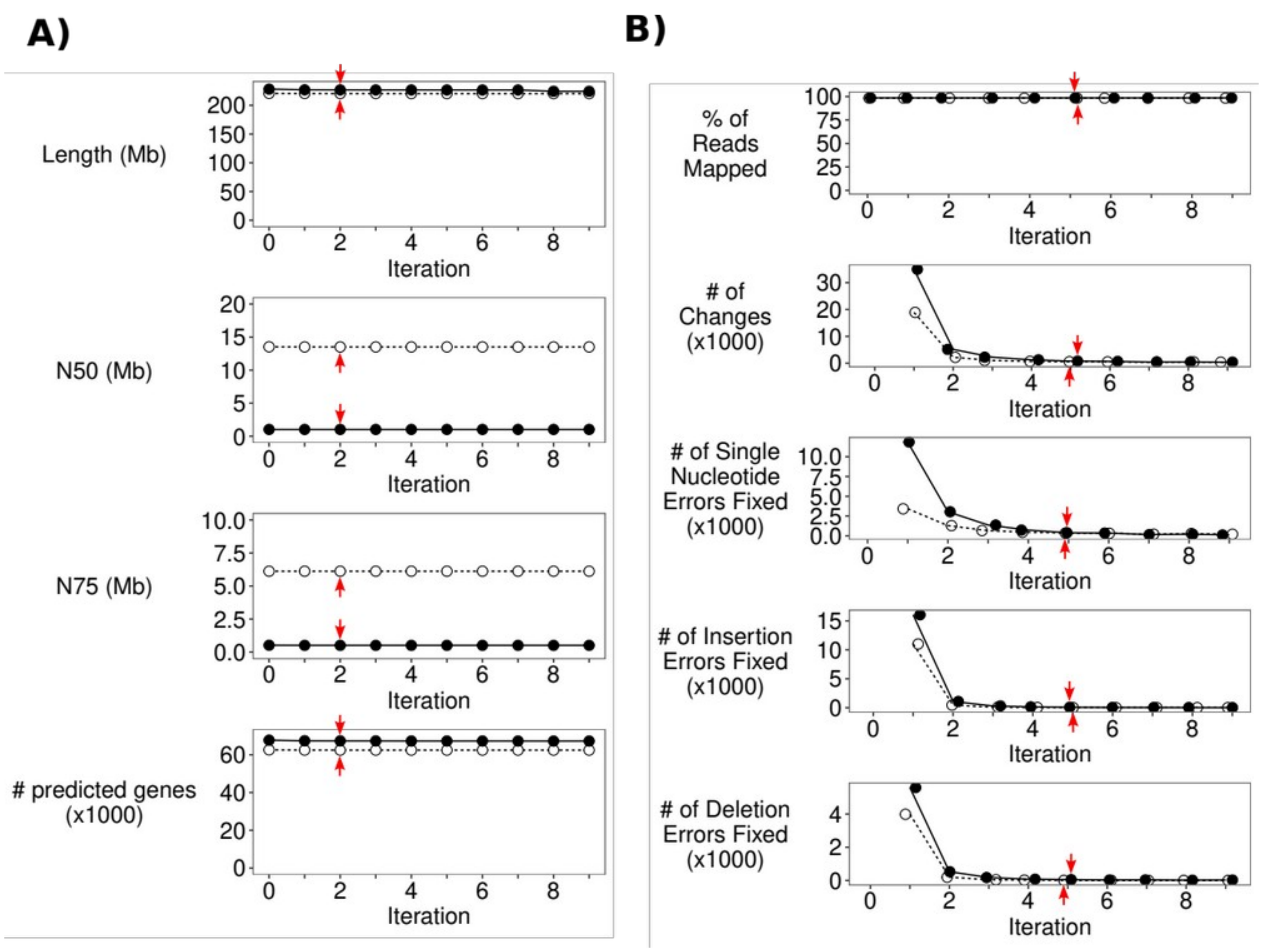

Figure S4. Summary statistics from successive iterations of polishing. A) polishing with Arrow/gcpp. Shown are (from top panel to bottom): the total length of the assembly, the N50 length of the assembly, the N75 length of the assembly, and the number of predicted genes in the assembly. **B)** polishing with pilon. Shown are (from top panel to bottom): The percentage of mapped Illumina reads, the total number of changes made to the assembly, the number of single nucleotide errors corrected, the number of insertion errors corrected, and the number of deletion errors corrected. In both panels, statistics are given for *M. cliftonense* (black circles) and *M. hystrix* (white circles). Red arrows show the iterations that were used for subsequent steps.

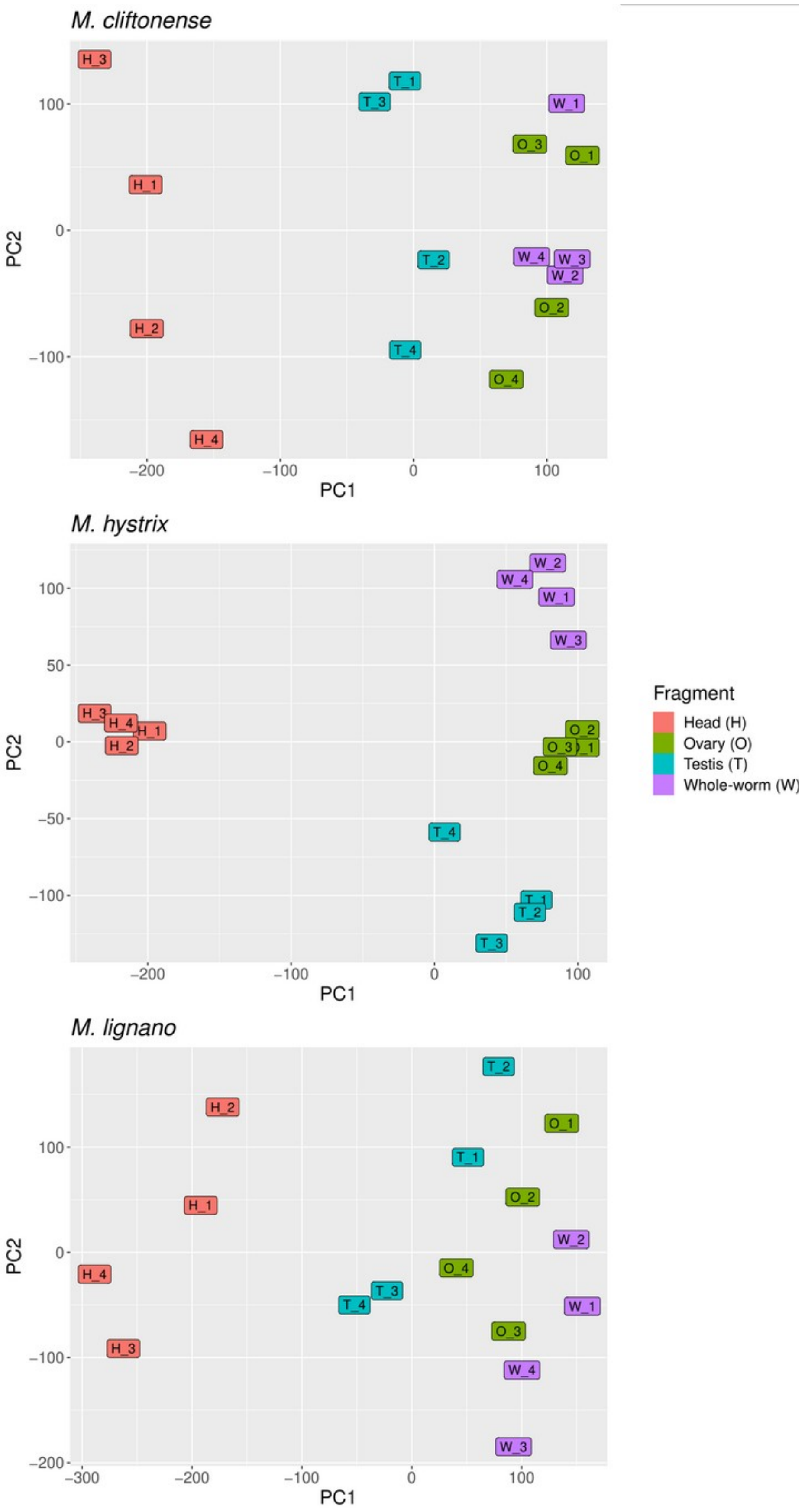

Figure S5. PCA plots for *M. cliftonense*, *M. hystrix*, and *M. lignano* of overall expression from all fragments of the positional RNA-Seq data-sets.

Figure S6. 3D-scatterplot of expression differences between neighbouring fragments for each transcript in the positional RNA-Seq for *M. cliftonense.* Points are coloured by their assigned positional annotation: blue – testis, orange – ovary 1, purple = ovary 2, green – tail, red – non-specific, black – other. See the supplementary file “figureS10_maccli_positional_3d.html”.

Figure S7. 3D-scatterplot of expression differences between neighbouring fragments for each transcript in the positional RNA-Seq for *M. hystrix.* Points are coloured by their assigned positional annotation: blue – testis, orange – ovary 1, purple = ovary 2, green – tail, red – non-specific, black – other. See the supplementary file “figureS11_machtx_positional_3d.html”.

Figure S8. 3D-scatterplot of expression differences between neighbouring fragments for each transcript in the positional RNA-Seq for *M. lignano.* Points are coloured by their assigned positional annotation: blue – testis, orange – ovary 1, purple = ovary 2, green – tail, red – non-specific, black – other. See the supplementary file “figureS12_maclig_positional_3d.html”.

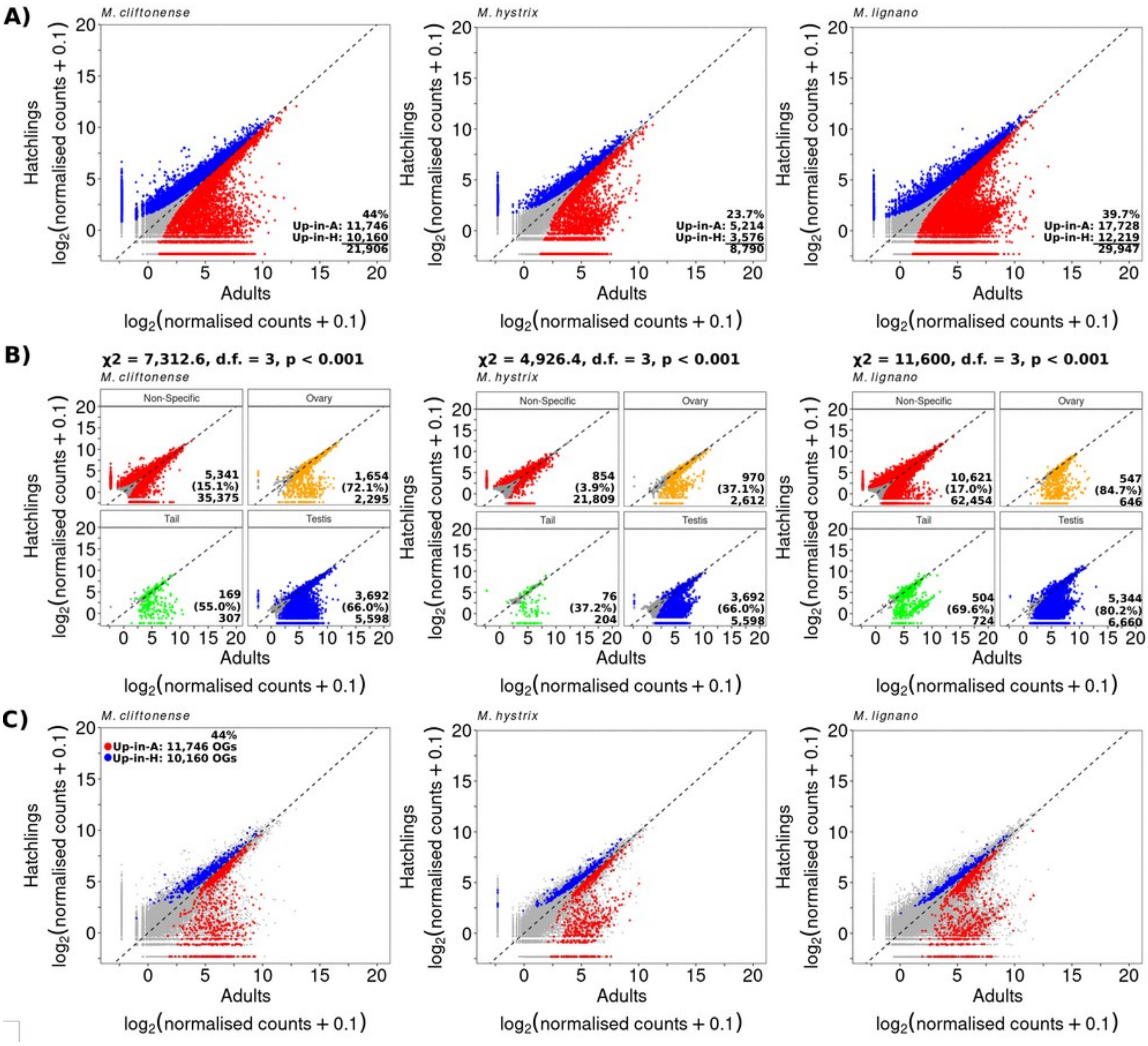

Figure S9. Mean log2 (normalised count) values across adults (*x*-axis) and hatchling (*y*-axis) pools for each species. In **A)**, red and blue points show transcripts with higher expression in adults (Up-in-A) and hatchlings (Up-in-H), respectively, within each species. The inset text gives the percentage of all transcripts that are differentially expressed in adult vs. hatchling worms, the absolute numbers of transcripts that are Up-in-A and Up-in-H, respectively, and the total number of transcripts that are differentially expressed. In **B)** transcripts are split by their positional annotation categories. Coloured points in each sub-panel are transcripts that are annotated as Up-in-A (below the diagonal) or Up-in-H (above the diagonal). Inset text in panels for each positional annotation category gives the number and percentage of the total in each category of transcripts that are Up-in-A, in each species. Also shown are the total number of transcripts with reproduction-related annotations. The text above each panel gives the results of a chi-squared test with expected proportions based on the proportion of analysed transcripts in each annotation category (table S9). In **C)**, red and blue points similarly show Up-in-A and Up-in-H transcripts that occur within one-to-one orthologs with the same adult vs. hatchling expression pattern across all three species.

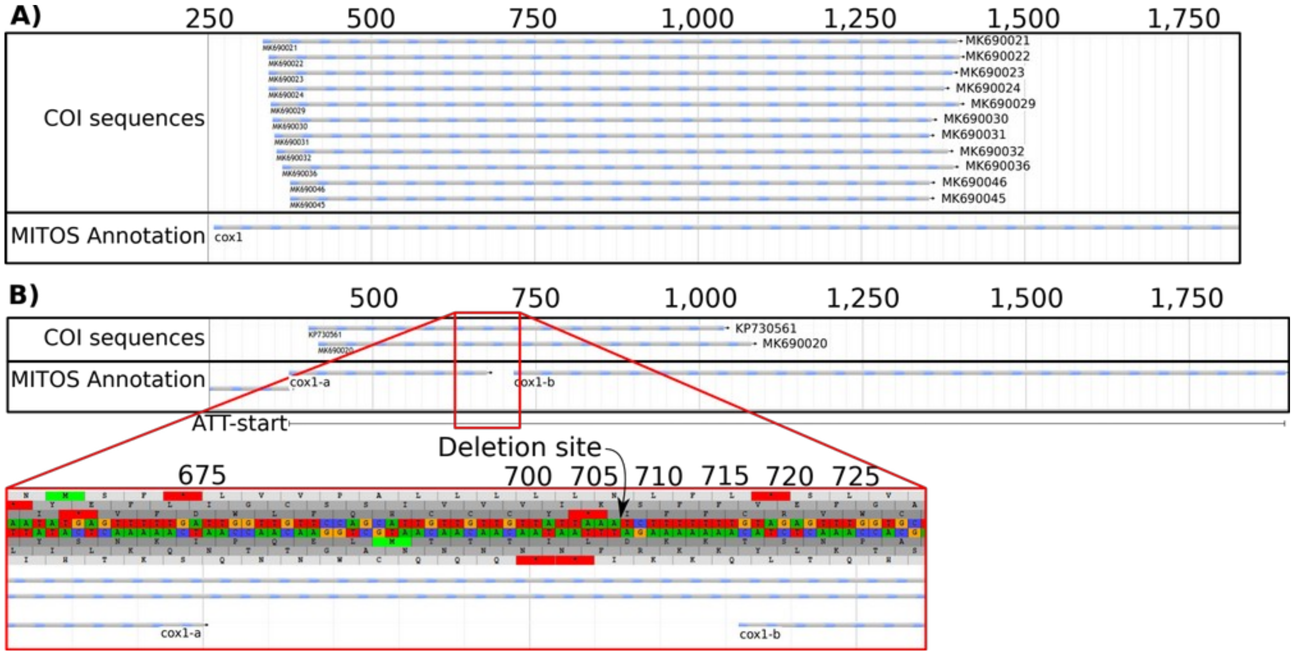

Figure S10. Mapping of partial COI sequences and the structure of MITOS2 gene annotations for regions of the mitochondrial genomes of **A)** *M. cliftonense* and **B)** *M. hystrix*. For *M. cliftonense*, in **A)**, a single contiguous locus (cox1) spanning all mapped partial COI sequences, labelled with their GenBank accession numbers, is annotated by MITOS2. In contrast, for *M. hystrix,* in **B)**, the annotated COI gene (cox1) is split in two (cox1-a and cox1-b). This split is very near the site of a previously described deletion (inset box), resulting in an AAT codon, and a premature STOP codon (TAG), 10 bases further downstream (Schärer et al. 2020). The reading frame in focus is the top amino acid sequence in the inset figure. Note that in flatworm mitochondrial genomes TGA (UGA) codons (as seen near position 675) codes for tryptophan instead of a translation STOP signal (translation tables 9 and 14). The JBrowse software, from which the figure is taken, only supports the standard genetic code by default.
